# Current insights and trends in atopic dermatitis and microbiota interactions: a systematic review and bibliometric analysis

**DOI:** 10.3389/fmicb.2025.1613315

**Published:** 2025-06-24

**Authors:** Zhongsong Zhang, Rong Wang, Mao Li, Mao Lu

**Affiliations:** ^1^Department of Dermatovenereology, Clinical Medical College, The First Affiliated Hospital of Chengdu Medical College, Chengdu, Sichuan, China; ^2^School of Clinical Medicine, Chengdu Medical College, Chengdu, China

**Keywords:** atopic dermatitis, microbiota, microbial transplantation, personalized treatment, bibliometric analysis

## Abstract

**Background:**

Atopic dermatitis (AD) is a prevalent chronic inflammatory skin condition influenced by immune dysfunction, genetics, and environmental factors, with emerging evidence highlighting the critical role of skin and gut microbiota in its pathogenesis. This article uniquely integrates a systematic review with bibliometric analysis to map the research landscape of AD and microbiota interactions, offering a comprehensive synthesis of trends and future directions.

**Methods:**

We conducted a bibliometric analysis using the Web of Science Core Collection, retrieving 1,196 English-language articles and reviews published between 2009 and 2024, employing a detailed search strategy targeting AD and microbiota-related terms. Data were analyzed with tools like CiteSpace, VOSviewer, and Biblioshiny to assess publication trends, geographical contributions, institutional outputs, journal impacts, author networks, reference citations, and keyword evolution.

**Results:**

Research on AD and microbiota has surged since 2016, peaking at 168 publications in 2021, with the USA leading in output (360 papers) and citations (24,655). The University of Copenhagen and the Journal of Allergy and Clinical Immunology emerged as top contributors, while authors like Gallo, Richard L., and Kong, Heidi H. drove influential studies. Key findings underscore the skin and gut microbiomes as research hotspots, with *Staphylococcus aureus* and the gut-skin axis dominating discussions. Emerging trends from 2020 to 2024 focus on adult AD severity, prebiotics, and personalized interventions like fecal microbiota transplantation (FMT), supported by multi omics data.

**Conclusion:**

This study illuminates the dynamic growth and global collaboration in AD and microbiota research, emphasizing microbial dysbiosis and immune modulation as pivotal to AD management. These insights pave the way for precision medicine and dietary interventions, promising enhanced therapeutic strategies and improved patient outcomes through continued multidisciplinary efforts.

## Introduction

Atopic dermatitis (AD) is a common chronic inflammatory skin disorder characterized by dry skin, erythema, and pruritus (Langan et al., 2020; Ständer, 2021). Recent studies have identified several factors that contribute to the development of AD, including immune system dysfunction, genetic predispositions, and environmental influences (Pareek et al., 2024; Vaseghi-Shanjani et al., 2025). Notably, increasing evidence points to the crucial role of the microbiota—especially the skin and gut microbiota—in the onset and progression of AD (Fang et al., 2021; Wrześniewska et al., 2024). In individuals with AD, the skin microbiota often exhibits dysbiosis, characterized by an overgrowth of pathogenic bacteria and a decline in beneficial microorganisms. This imbalance may exacerbate immune dysfunction and fuel the inflammatory response in the skin (Paller et al., 2019).

**Figure 1:**
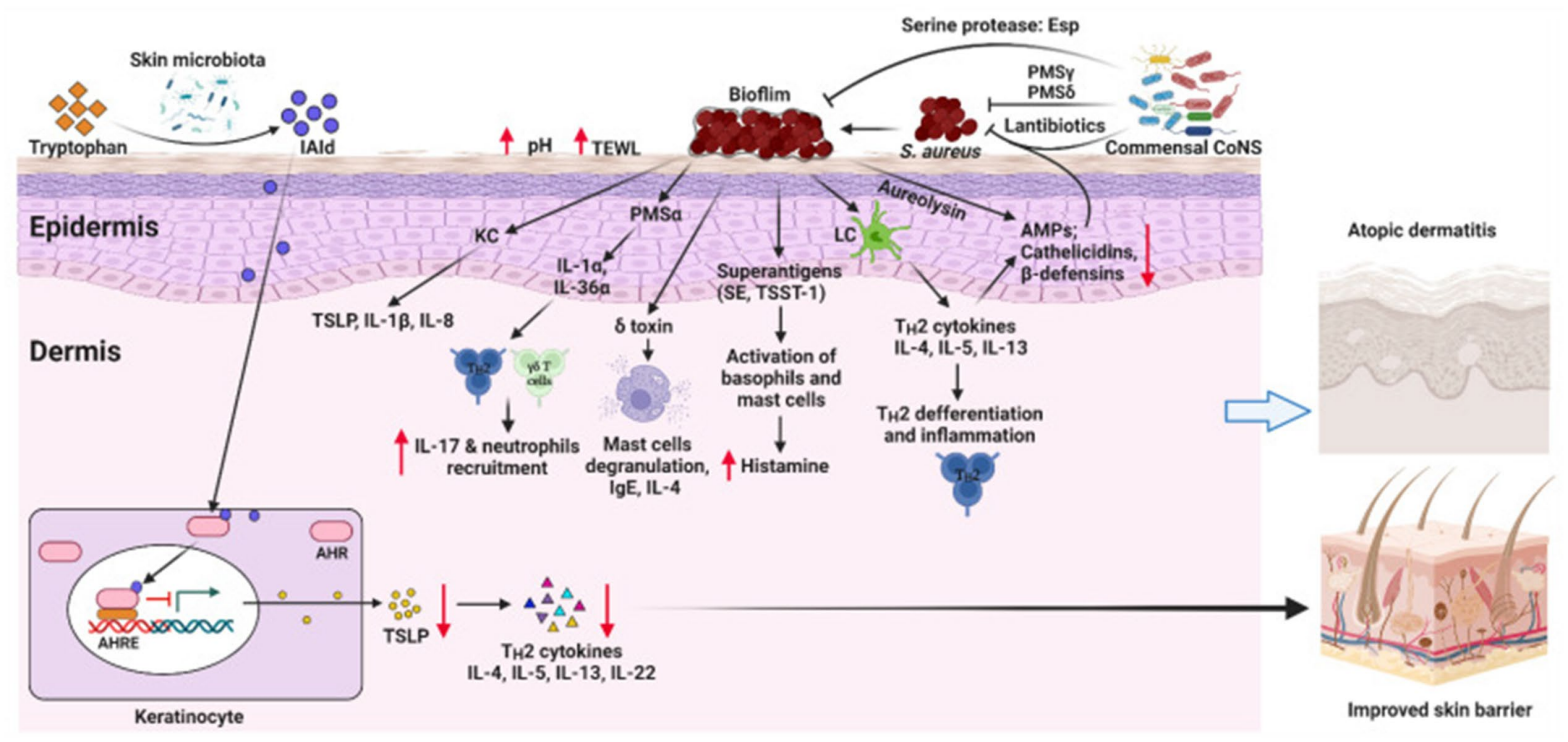
This schematic illustrates the interplay between skin microbiota dysbiosis and AD development. Dysbiosis in the skin microbiota, characterized by an overgrowth of pathogenic bacteria (e.g., *S. aureus*) and a reduction in beneficial microorganisms (e.g., Commensal CoNS), disrupts the skin barrier and increases pH and transepidermal water loss (TEWL). This leads to the release of alarmins (e.g., TSLP, IL-1*β*, IL-8) by keratinocytes, which recruit immune cells such as IL-17 + neutrophils and activate Th2 cytokines (IL-4, IL-5, IL-13, IL-22), driving inflammation and immune dysfunction in the epidermis and dermis. Superantigens (SE, TSST-1) and toxins from *S. aureus* further exacerbate inflammation by activating basophils and mast cells, releasing histamine. The microbiota-immune axis is highlighted, where dysbiosis influences skin inflammation through immune pathways, contributing to AD symptoms. Restoring microbial balance with beneficial bacteria (e.g., Commensal CoNS) and reducing pathogenic load (e.g., through AMPs, cathelicidins, *β*-defensins) may improve skin barrier function and mitigate AD severity, offering new perspectives for treatment strategies. AHR, aryl hydrocarbon receptor; AHRE, AHR element; AMPs, antimicrobial peptides; CoNS, Coagulase-negative staphylococci; IAId, indole-3-aldehyde; IL, interleukin; ILC3, lymphoid cells type 3; KC, keratinocyte; LC, Langerhans cell; PSM, phenol-soluble modulin, TSLP, thymic stromal lymphopoietin; SE, Staphylococcal enterotoxin; TSST-1, toxic shock syndrome toxin-1. Reproduced from Alam, Md Jahangir et al. Manipulating Microbiota to Treat Atopic Dermatitis: Functions and Therapies. Pathogens (Basel, Switzerland). Copyright © 2022 by Alam et al. (2022).

The skin microbiota is essential for regulating immune responses and maintaining the skin’s barrier function (Liang et al., 2021). Disruption of the skin barrier and overactivation of immune responses are strongly linked to the development of AD (Li et al., 2021), processes that may be influenced by the composition of the skin microbiota (Figure 1) (Hrestak et al., 2022). Similarly, alterations in the gut microbiota have also been associated with the pathogenesis and severity of AD (Lv et al., 2022; Mohammad et al., 2024). Studies have highlighted the existence of a microbiota-immune axis between the gut and skin (Luger et al., 2021), where dysbiosis in the gut microbiota can influence skin inflammation through immune pathways, exacerbating AD symptoms (Hrestak et al., 2022). The relationship between AD and the microbiota has become a major focus of research, particularly regarding the regulatory role of the skin microbiota in immune function and AD pathogenesis (Grice and Segre, 2011; Edslev et al., 2020). Despite considerable investigation into the microbiota’s potential role in AD, research in this area remains fragmented and lacks a comprehensive synthesis (Chng et al., 2016). Therefore, a bibliometric analysis is needed to consolidate existing studies and clarify the current research trends and future directions in this field (Wang et al., 2023; Farnetano et al., 2024).

This study is the first to reveal potential directions for AD and microbiota research through a multidimensional bibliometric analysis and thematic modeling. Compared to traditional analyses, this paper provides a more comprehensive knowledge graph and interdisciplinary perspective (Jin et al., 2023; Wang Y. et al., 2024, pp. 2014–2023). Furthermore, it seeks to offer new perspectives for advancing AD treatment strategies.

## Materials and methods

### Data collection

Our bibliometric analysis is anchored in the Web of Science Core Collection (WOSCC), widely recognized as the preeminent and most authoritative dataset for bibliometric research. The WOSCC provides an extensive array of data fields, enabling a comprehensive and in-depth analytical approach. Leveraging this robust dataset, the data retrieval process was meticulously designed to ensure both accuracy and completeness, with two researchers independently conducting the retrieval and implementing dual verification procedures. The selection and extraction of documents pertinent to the study were similarly performed independently by the two researchers and subjected to rigorous evaluation against predefined inclusion and exclusion criteria. To further safeguard the integrity of the data, any discrepancies or inconsistencies arising during the retrieval process were resolved through consultation with a third researcher, who conducted a thorough review and facilitated discussion, thereby upholding the reliability and impartiality of the research outcomes.

**Figure 2:**
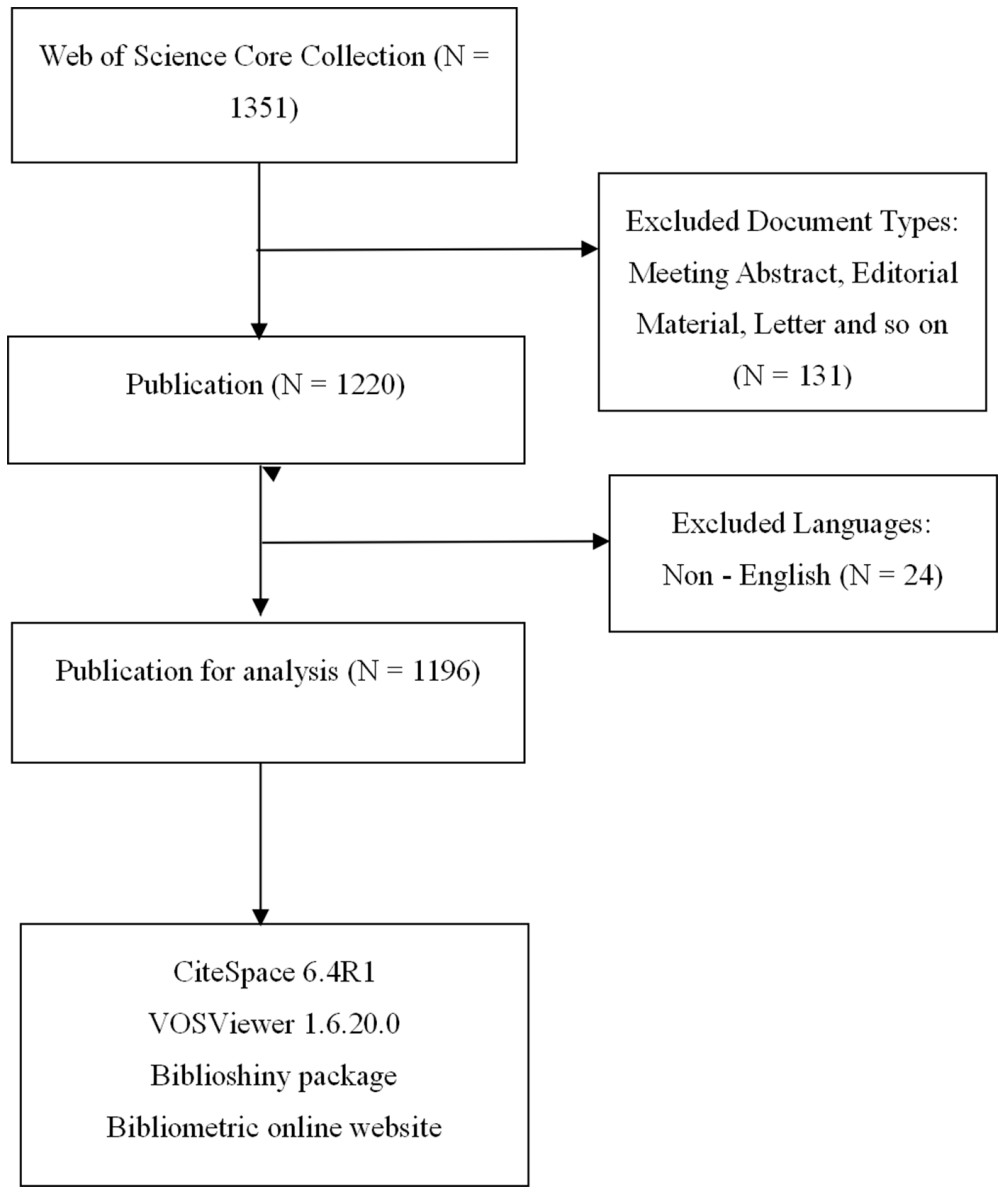
Flow chart depicting data collection strategies for AD and microbiota.

We conducted a comprehensive bibliometric analysis by designing a search formula that included a broad range of terms related to AD and microbiota: “(TS = (“Atopic Dermatitis” OR “Eczema, Atopic” OR “Atopic Eczema” OR “Neurodermatitis, Atopic” OR “Atopic Neurodermatitis” OR “Neurodermatitis, Disseminated” OR “Disseminated Neurodermatitis” OR “Eczema, Infantile” OR “Infantile Eczema” OR “Dermatitis, Atopic”) AND TS = (“Microbiotas” OR “Microbial Community” OR “Community, Microbial” OR “Microbial Communities” OR “Microbial Community Composition” OR “Community Composition, Microbial” OR “Composition, Microbial Community” OR “Microbial Community Compositions” OR “Microbiome” OR “Microbiomes” OR “Human Microbiome” OR “Human Microbiomes” OR “Microbiome, Human” OR “Microbial Community Structure” OR “Community Structure, Microbial” OR “Microbial Community Structures”)).” To maintain focus and quality, we restricted the eligible publication types to articles and reviews published in English. This search yielded 1,196 relevant documents published between January 1, 2009, and December 31, 2024, which were selected for inclusion based on their alignment with our research criteria. The results were subsequently recorded in plain text format, capturing the full records and cited references for detailed evaluation (Figure 2).

### Data analysis

We compiled data on publications, document types, countries/regions, institutions, authors, journals, references, and keywords from the retrieved literature. During this process, we removed spelling errors and consolidated identical author names and synonymous keywords to ensure data accuracy. The processed data were then imported into several tools for bibliometric and visualization analysis, including CiteSpace, VOSviewer, Microsoft Excel 365, the Biblioshiny package, and a bibliometric analysis website. Each of these tools serves a specific purpose in our analysis. Specifically, CiteSpace is a Java-based application that provides interactive visualization tools for bibliometric analysis by integrating bibliometrics, visualization methods, and data mining algorithms (Chen, 2006). Similarly, VOSviewer focuses on creating bibliometric network maps to visualize relationships within the data (van Eck and Waltman, 2010). The Biblioshiny package, a web-based interface for the R-based Bibliometrix package, utilizes the Shiny framework to offer interactive tools for bibliometric analysis, enabling users without coding experience to import data, generate networks, and create trend visualizations. Additionally, bibliometric analysis website, such as the Bibliometrix website, provides similar functionalities, allowing users to upload data, analyze literature, and visualize results directly through a web browser without the need for local software installation.

We employed CiteSpace, VOSviewer, and the Biblioshiny package to conduct a comprehensive bibliometric analysis of research on AD and microbiota. Specifically, we analyzed the geographical distribution of this research across different countries and regions, and examined the institutions, authors, and their collaboration networks. Furthermore, we investigated co-citation patterns, detected emerging trends in the literature, identified rapidly increasing keywords, studied the evolution of terms over time, and assessed publication and citation trends. These analyses enabled us to understand the overall scope and developmental trajectory of academic research in this field. By uncovering foundational research patterns and illustrating the structure and evolution of the field, we are better equipped to forecast future research directions and pinpoint emerging hot topics.

## Results

### Publication output and temporal trend

**Figure 3:**
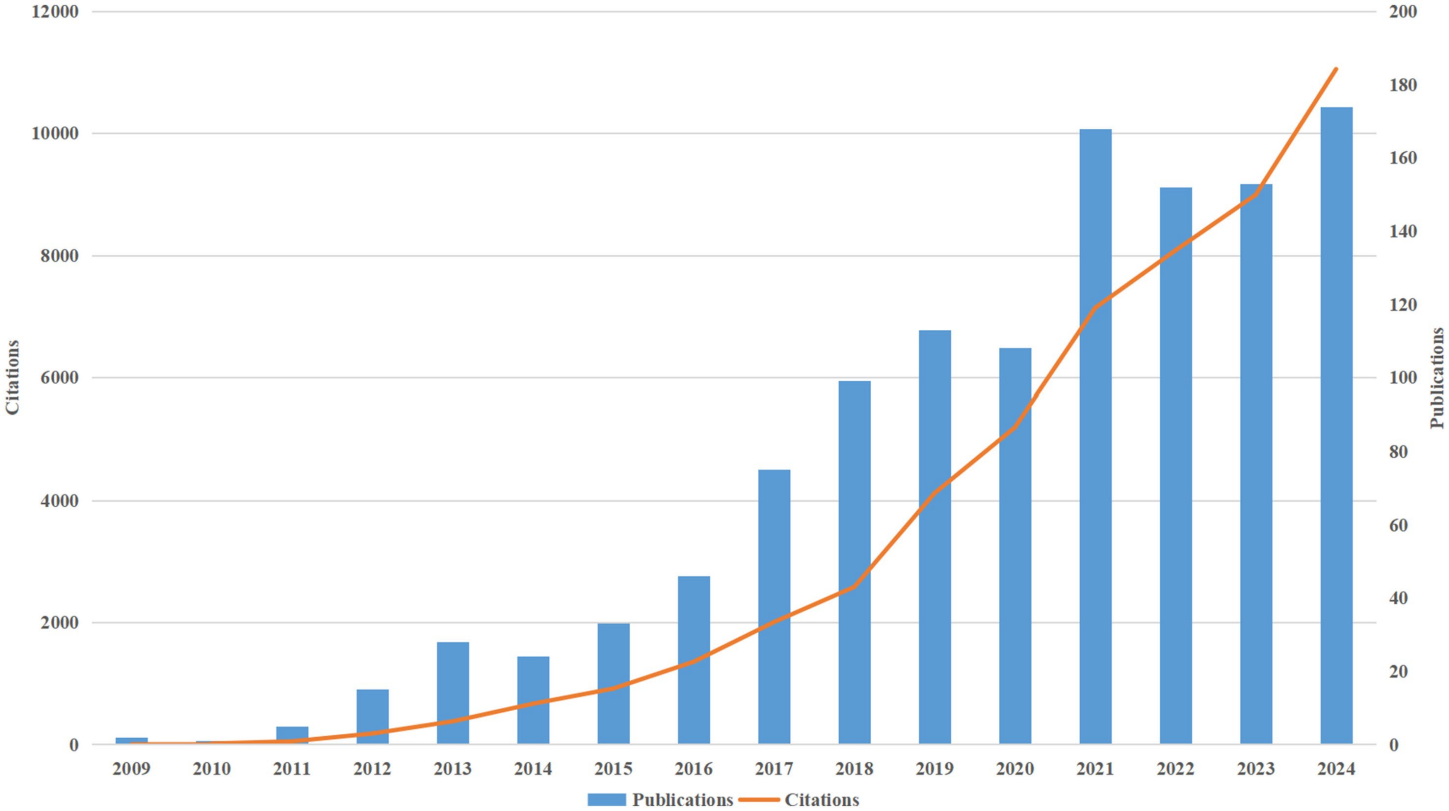
Shows a consistent rise in the annual publications and citations of research on AD and microbiota from 2009 to 2024.

Since 2009, research on AD and microbiota has been published, with both publication and citation volumes showing a significant upward trend. Between 2009 and 2015, publication volume remained relatively low; however, from 2016 onwards, there was a noticeable acceleration in the publication rate. This growth became especially pronounced after 2020, temporarily peaking at 168 publications in 2021. This upward trend highlights the increasing attention that the field of AD and microbiota has received from researchers over time (Figure 3). Concurrently, we observed that the growth in publication and citation volumes has been closely aligned, with citations increasing at a faster pace than publications. This suggests that research on AD and microbiota has had a growing impact in the academic community. Additionally, the continued rise in both citations and publications points to the likely need for more forward-looking research to further deepen our understanding of AD and microbiota, ultimately driving scientific progress.

### Analysis of countries/regions

**Figure 4:**
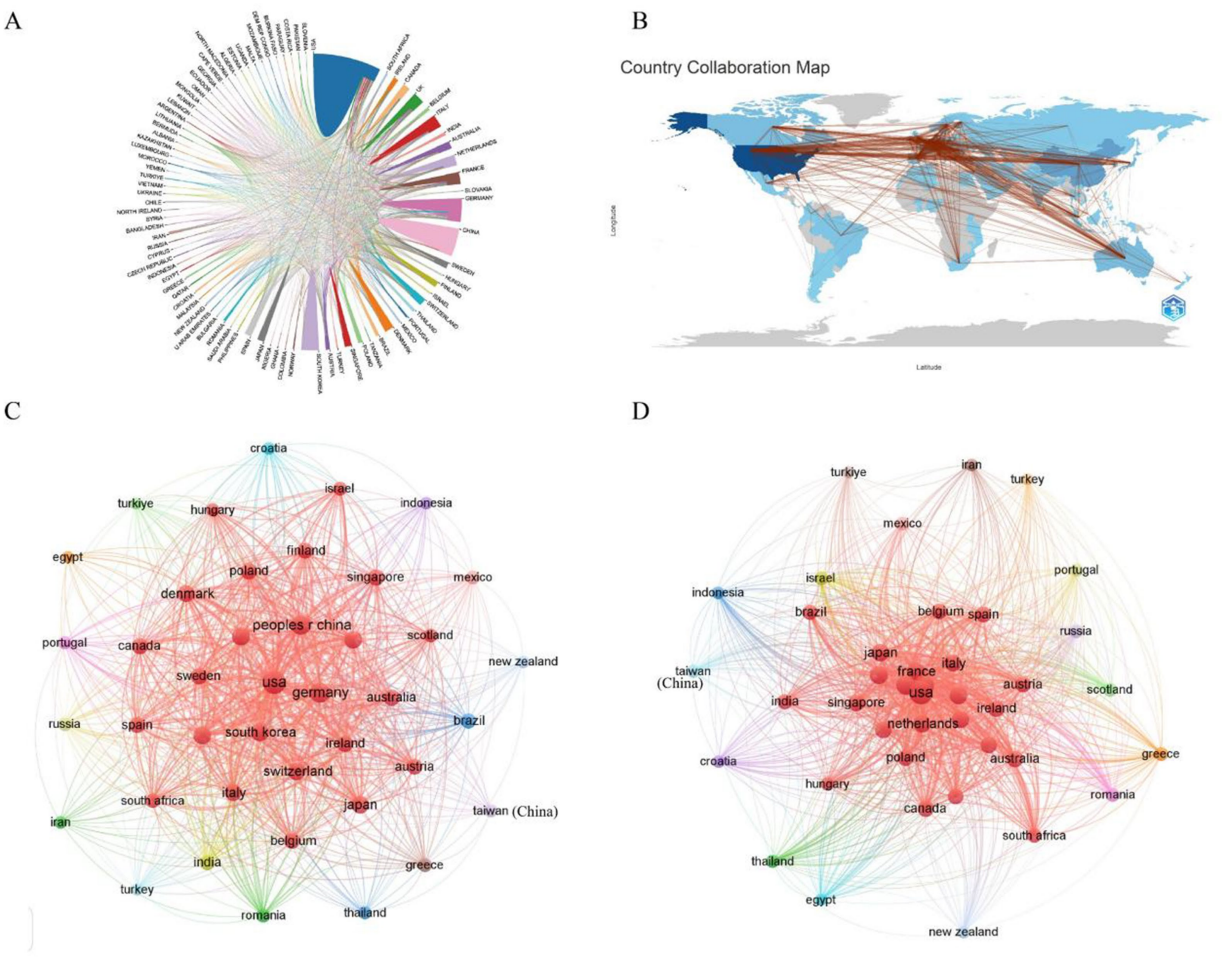
**(A)** A co-occurrence map illustrating collaboration between countries/regions in AD and microbiota research. The size of each circle corresponds to the volume of publications from a country/region, while the thickness of the connecting lines indicates the strength of collaboration. **(B)** A network map depicting international collaborations in the field. The lines represent cooperative relationships between countries/regions. **(C)** A bar chart showing the number of citations of AD and microbiota-related publications by various countries/regions. **(D)** A citation coupling map identifying countries that cite similar research articles, revealing shared academic interests and thematic collaborations within the field.

As of 2024, a total of 70 countries or regions have contributed to research on AD and microbiota (Figure 4A). Among them, we ranked the top 10 countries/regions with the highest number of publications on this topic (Table 1). The United States leads by a significant margin, with 360 publications. Both Germany (123) and China (111) have also published over 100 articles related to AD. It is worth noting that the United States has accumulated 24,655 citations, far surpassing other countries. Although China ranks second in total citations, the U. S. citation count is approximately five times higher, indicating the dominant influence of the United States in both the quantity and quality of research output. From Figure 4B, it is evident that international collaborations are primarily concentrated in the Northern Hemisphere. Notably, Europe demonstrates the most extensive collaborative networks, including connections with countries in the Southern Hemisphere. This underscores Europe’s pivotal role in advancing AD and microbiota research. Furthermore, countries with a higher number of publications tend to engage in closer collaborations, as demonstrated by the strong research exchange between China and the United States (Figures 4C,D).

**Table 1:** The top 10 productive countries with publications concerning AD and microbiota.

Rank	Country	Documents	Citations	Total link strength
1	USA	360	24,655	326
2	Germany	123	4,900	259
3	China	111	3,351	64
4	south Korea	76	2,315	20
5	France	68	2,878	123
6	Netherlands	59	3,350	169
7	England	58	2,740	177
8	Japan	57	1738	38
9	Switzerland	53	2,372	160
10	Italy	48	1727	110

### Analysis of institutions

**Figure 5:**
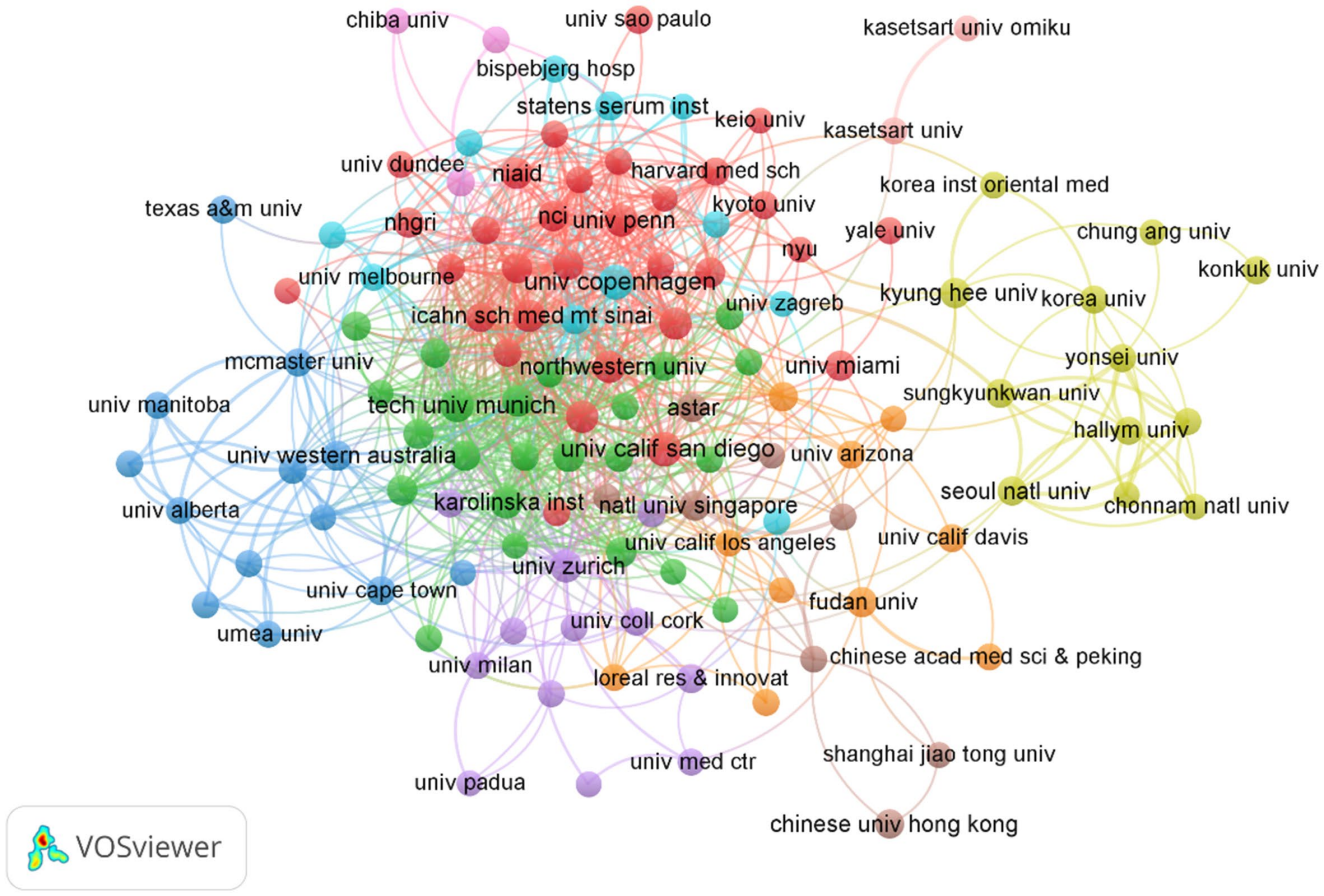
Cluster analysis of institutions related to AD and microbiota. Each node represents an institution, with the size of the circle corresponding to the number of publications from that institution.

Table 2 illustrates the top 10 most productive institutions. The University of Copenhagen published 31 papers and was the institution with the greatest contribution to this area, followed by University of California, San Diego (30), the Technical University of Munich (29). From the institution collaboration map and the associated data (Figure 5), it is evident that several renowned universities and research institutions play key roles in AD and microbiota research. Institutions such as the University of Copenhagen, University of California, San Diego, and Technical University of Munich are leading in terms of both the number of publications and their impact in the field. From the data and visualization, it is clear that the AD and microbiota research field is a globally collaborative and densely connected domain, with major universities and research institutions in USA and Europe at its core, significantly advancing both the quantity and quality of research in this area.

**Table 2:** The top 10 productive research institutions with publications concerning AD and microbiota.

Rank	Organization	Original country	Documents	Citations	Total link strength
1	Univ Copenhagen	Denmark	31	1831	51
2	Univ Calif San Diego	USA	30	2,795	46
3	Tech Univ Munich	Germany	29	1,086	81
4	Icahn Sch Med Mt. Sinai	USA	23	2,485	69
5	Natal Jewish Health	South Africa	20	2,108	60
6	Univ Colorado	USA	20	1,601	66
7	Karolinska Inst	Sweden	19	1,592	50
8	Northwestern Univ	USA	19	909	45
9	Univ Penn	USA	19	1,498	40
10	Univ Zurich	Switzerland	19	1,229	54

### Analysis of journal

We conducted a visual analysis of publishing and co-cited journals to identify the most active and influential sources related to AD and microbiota. A total of 1,196 publications on this topic were published across 327 academic journals. Among these, The Journal of Allergy and Clinical Immunology (IF 11.4) and Allergy (IF 12.6) emerged as the most frequently publishing and co-cited journals (Table 3).

**Table 3:** The top 10 productive academic journals with publications concerning AD and microbiota.

Rank	Journal	Documents	IF (JCR2024)	JCR quartile	Citations
1	Journal of allergy and clinical immunology	48	11.4	Q1	5,561
2	Allergy	39	12.6	Q1	1,520
3	International journal of molecular sciences	30	4.9	Q2	921
4	Frontiers in immunology	28	5.7	Q2	889
5	Microorganisms	23	4.1	Q2	937
6	Experimental dermatology	21	3.5	Q2	689
7	Journal of investigative dermatology	19	5.7	Q2	840
8	Journal of drugs in dermatology	17	1.5	Q4	368
9	Nutrients	17	4.8	Q2	458
10	Allergy asthma & immunology research	15	4.1	Q2	770

**Figure 6:**
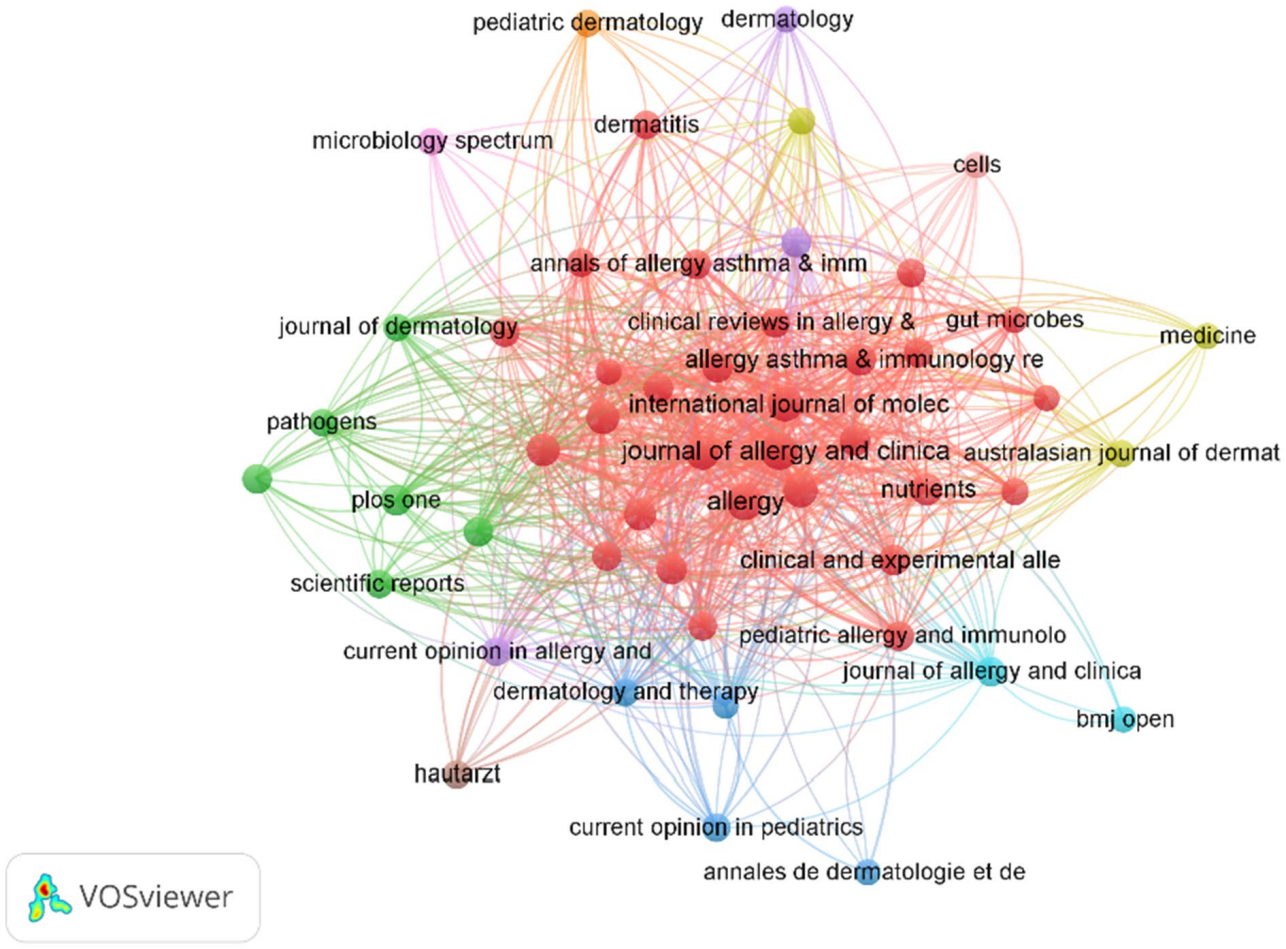
A visualization of the relationship between co-cited journals. Each node represents a journal, with the node size corresponding to its citation frequency, and the size reflecting the number of co-citations.

In addition, based on co-citation frequency analysis (Figure 6), journals are grouped into four clusters. First, the Allergy, Asthma & Immunology Zone includes core journals such as Annals of Allergy, Asthma & Immunology, Journal of Allergy and Clinical Immunology, and Pediatric Allergy and Immunology. These nodes, predominantly colored red, form a densely interconnected cluster, contributing the majority of articles on topics related to allergy, asthma, and immunology. Second, the Dermatology Zone comprises journals such as Dermatology, Pediatric Dermatology, and Journal of Dermatological Science, primarily distinguished by orange and purple hues. This journal focuses on research pertaining to dermatological conditions, with the curve gradually flattening. Third, the Microbiology & Pathogens Zone encompasses journals like Microbiology Spectrum and Pathogens, marked by green nodes, and addresses studies on microbiology and gut microbiot. Finally, the other fields zone includes journals such as Medicine, Nutrients, and Cells, identified by yellow or other colors, covering broader medical or biological research These journals play a central role in AD and microbiome research, facilitating academic collaboration and progress, and establishing a solid foundation for future research in the field.

### Analysis of authors

**Figure 7:**
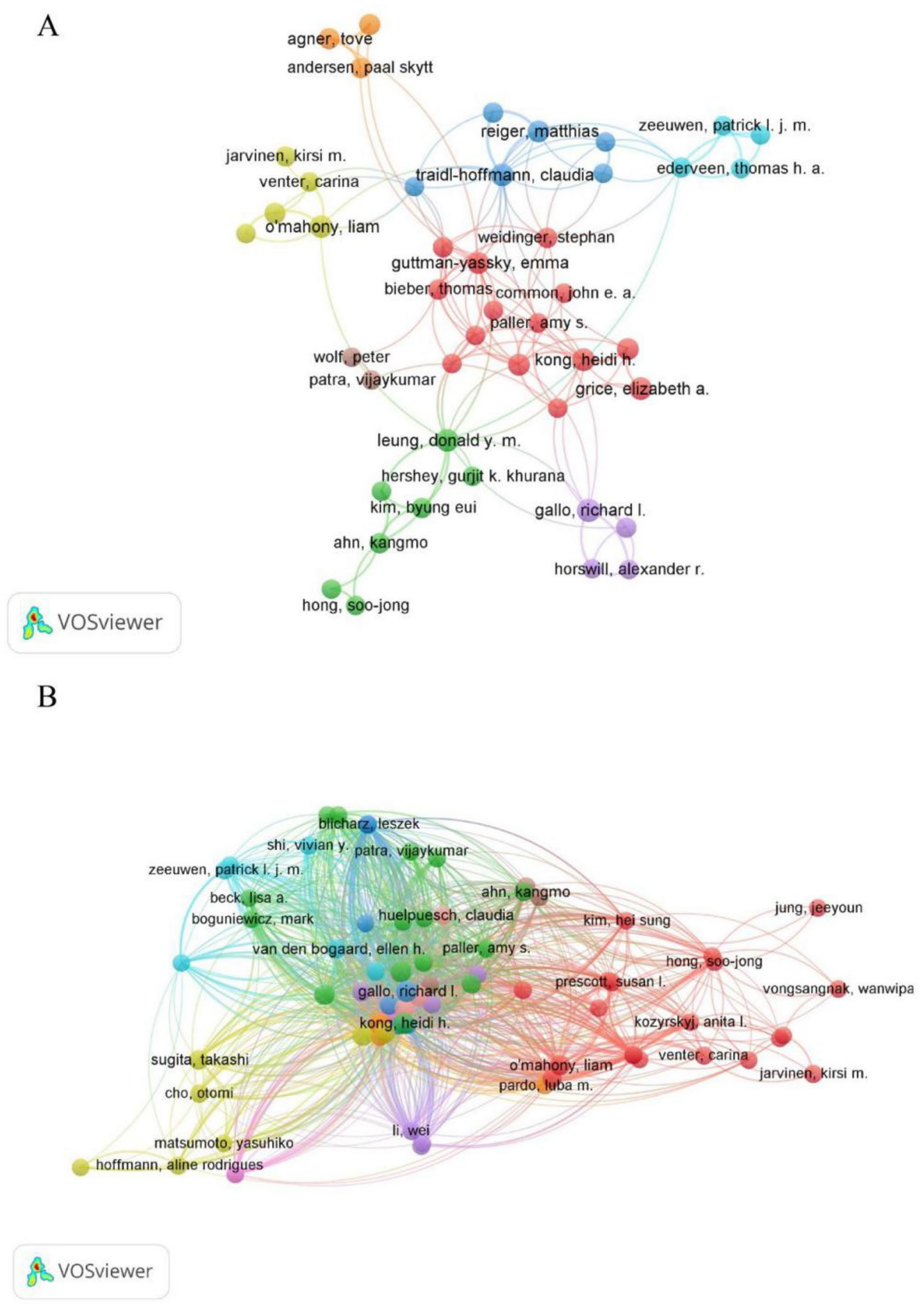
Author-level analysis in AD and microbiota research. **(A)** A co-authorship map illustrating authors involved in AD and microbiota studies. Node colors represent distinct collaboration clusters, node size reflects the frequency of co-authorship, and links indicate co-authorship relationships. **(B)** A co-citation map of authors, where node size denotes citation frequency, revealing influential researchers based on how often they are cited together. Data were visualized using VOSviewer.

A total of 5,078 authors participated in the research of AD and microbiota. Table 4 shows the top 10 most prolific authors in AD and microbiota study. Gallo, Richard L published 17 papers with 2,526 citations, is a renowned expert in the field of skin microbiology, with a research focus on the relationship between skin antimicrobial peptides and microbiota with AD. showing its central position in AD and microbial research. Kong, Heidi H., as a distinguished scholar in the fields of AD and microbiology, although ranks second with 16 published articles but with 3,610 citations, underscoring her substantial academic influence (Table 4). Interestingly, Andrew N. J. McKenzie and Foo Yew Liew (despite not appearing in the Table 4) are likely positioned at the center of the network, bridging multiple clusters, which suggests their pivotal role as connectors in AD and microbiology research. The cross-cluster collaborations in Figures 7A,B, such as the connections between different clusters, suggest that future research could further enhance multidisciplinary integration of AD and microbiology in different author’s filed. Overall, the authorship and collaboration networks in the fields of AD and microbiology demonstrate a high degree of concentration and synergy. Authors such as Gallo, Richard L., Kong, Heidi H., and Leung, Donald Y. M. are core contributors, whose high productivity and extensive collaborations (within the red, green, and blue clusters in Figure 7A) have advanced progress in these fields.

**Table 4:** Top 10 authors in terms of number of publications.

Rank	Author	Documents	Citations	Total link strength
1	Gallo, Richard L.	17	2,526	23
2	Kong, Heidi H.	16	3,610	22
3	Leung, Donald Y. M.	15	1927	28
4	Traidl-hoffmann, Claudia	14	891	32
5	Grice, Elizabeth A.	14	4,462	5
6	O’mahony, Liam	13	636	16
7	Segre, Julia A.	12	6,314	15
8	Guttman-yassky, Emma	12	1,142	22
9	Reiger, Matthias	11	170	24
10	Irvine, Alan D.	11	1,470	22

### Analysis of reference

The references listed in Table 5 are the top 10 highly cited references, published between 2009 and 2018, which have accumulated citations ranging from 104 to 385, reflecting their profound impact on understanding the role of microbiota in the pathology of AD and skin health. “Temporal shifts in the skin microbiome associated with disease flares and treatment in children with atopic dermatitis” published in Genome Res in 2012, is the most highly cited, which stands as one of the most influential publications, employing metagenomic sequencing to explore the human skin microbiome. Then followed by “Antimicrobials from human skin commensal bacteria protect against *Staphylococcus aureus* and are deficient in atopic dermatitis,” “Topographical and Temporal Diversity of the Human Skin Microbiome.”

**Table 5:** Top 10 highly cited references.

Rank	Cited Reference	DOI	Citations	Author	Journal	Year
1	Temporal shifts in the skin microbiome associated with disease flares and treatment in children with atopic dermatitis	doi 10.1101/gr.131029.111	385	Heidi H Kong	Genome Res	2012
2	Antimicrobials from human skin commensal bacteria protect against *Staphylococcus aureus* and are deficient in atopic dermatitis	doi 10.1126/scitranslmed.aah4680	194	Teruaki Nakatsuji	Sci Transl Med	2017
3	Topographical and temporal diversity of the human skin microbiome	doi 10.1126/science.1171700	191	Elizabeth A Grice	Science	2009
4	The skin microbiome	doi 10.1038/nrmicro2537	186	Elizabeth A Grice	Nat Rev. Microbio	2011
5	*Staphylococcus aureus* and *Staphylococcus epidermidis* strain diversity underlying pediatric atopic dermatitis	doi 10.1126/scitranslmed.aal4651	145	Allyson L Byrd	Sci Transl Med	2017
6	The human skin microbiome	doi 10.1038/nrmicro.2017.157	143	Allyson L Byrd	Nat Rev. Microbiol	2018
7	Skin microbiome before development of atopic dermatitis: Early colonization with commensal staphylococci at 2 months is associated with a lower risk of atopic dermatitis at 1 year	doi 10.1016/j.jaci.2016.07.029	127	Elizabeth A Kennedy	J Allergy Clin Immunol	2017
8	Low diversity of the gut microbiota in infants with atopic eczema	doi 10.1016/j.jaci.2011.10.025	119	Thomas R Abrahamsson	J Allergy Clin Immunol	2012
9	Delivery mode shapes the acquisition and structure of the initial microbiota across multiple body habitats in newborns	doi 10.1073/pnas.1002601107	112	Maria G Dominguez-Bello	Proc Natl Acad Sci U S A	2010
10	Dysbiosis and *Staphylococcus aureus* colonization drives inflammation in atopic dermatitis	doi 10.1016/j.immuni.2015.03.014	104	Tetsuro Kobayashi	Immunity	2015

**Figure 8:**
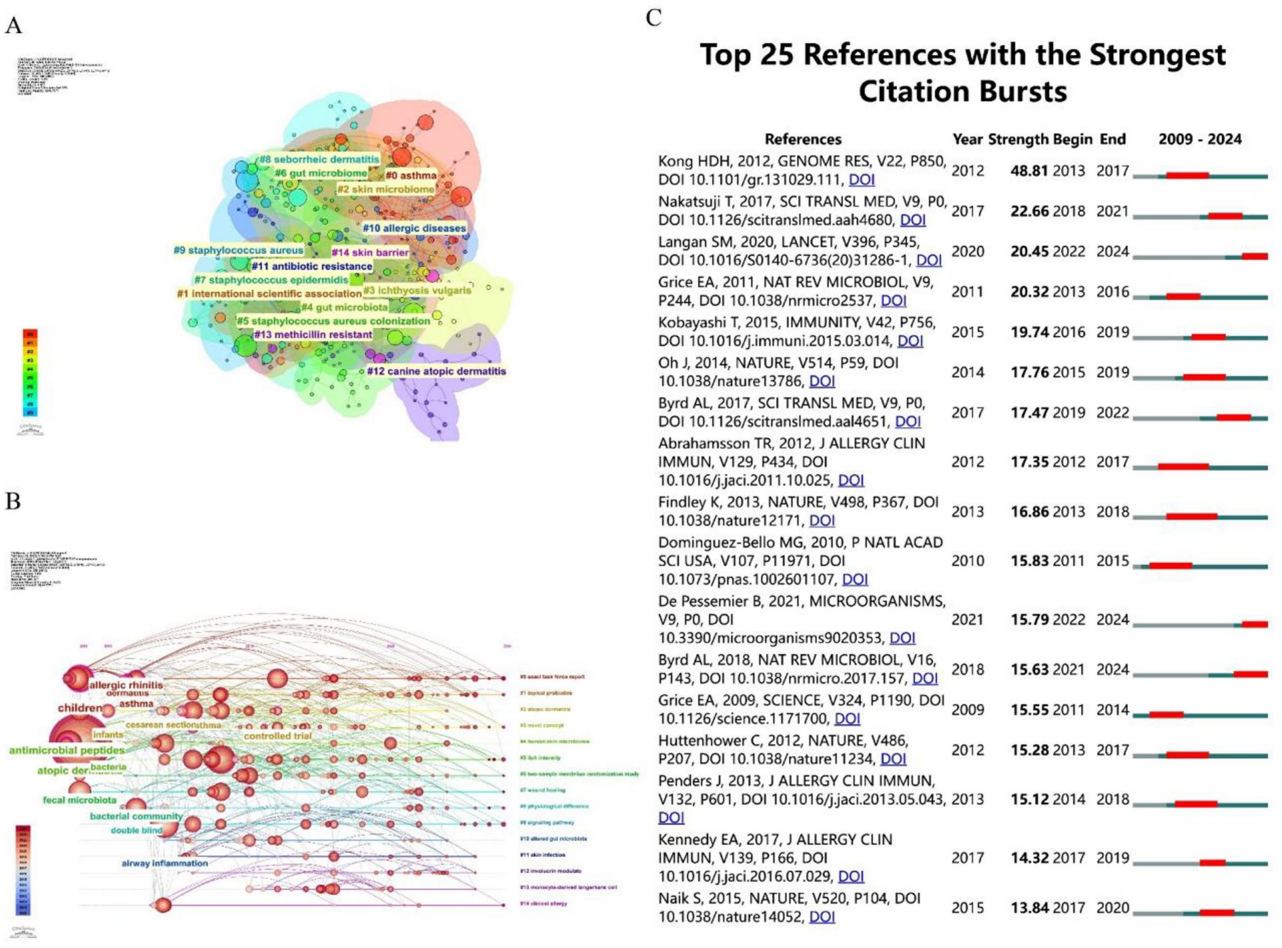
**(A)** The clustering of literature within the literature relationship network graph, consisting of a total of 15 clusters. **(B)** A timeline visualization map generated by CiteSpace, illustrating the literature clustering related to AD, highlighting the first appearance of each key keyword over time. **(C)** The top 25 pieces of literature with the highest burst in citations. The blue line represents the timeline, while the red phase indicates the outbreak period. This visualization is based on a burst test used to detect sudden changes in information, such as documents and keywords.

Figure 8A displays a keyword co-occurrence network, with clusters representing major research themes related to AD and microbiolog. The size of the nodes indicates the frequency or importance of each keyword, while the connecting lines show the strength of their co-occurrence. The color gradient reflects the temporal distribution of research activity, with red representing earlier bursts (2009–2014) and blue indicating more recent activity (2019–2024). Key clusters and their relevance to AD and microbiology are below: #1 International scientific association, #2 Skin microbiome, #4 Gut microbiota, #9 *Staphylococcus aureus*, #7 *Staphylococcus epidermidis*: These clusters are central to AD research, aligning with the findings in the Table 5 and previous analyses. Figure 8B presents a timeline view of citation bursts for key references and themes from 2009 to 2024. The timeline shows a progression from foundational microbiome studies to applied research on microbial-immune interactions, therapeutic interventions (e.g., probiotics, antimicrobial peptides), and clinical outcomes (e.g., skin barrier, allergic diseases). Topics like “topical probiotics” (#1), “atopic dermatitis” (#2), and “skin microbiome” (#4) are recurrent, aligning with the keyword co-occurrence findings. Figure 8C illustrates the top 25 references with the strongest citation bursts. During the period from 2009 to 2024, citation bursts exhibited a diverse distribution pattern, with one or more bursts potentially occurring each year, typically lasting several years. The year 2015 stands out as a significant peak, with multiple references initiating citation bursts, some of which extended into 2018 or beyond. For instance, the article by Oh J, published in Genome Research in 2013, experienced a burst from 2014 to 2017. Notably, 2015 witnessed up to six citation burst events (e.g., Naik et al., 2015; Kobayashi et al., 2015), with most persisting until 2018 or 2019, marking the highest frequency of bursts in recent years. These highly cited references collectively underscore the critical role of skin and gut microbiota in AD, highlighting the significance of microbial dysbiosis, immune modulation, and therapeutic potential. They delineate a multidisciplinary research trajectory integrating microbiology, immunology, and dermatology, providing a robust foundation for advancing the management and prevention strategies for AD.

### Analysis of keywords

**Figure 9:**
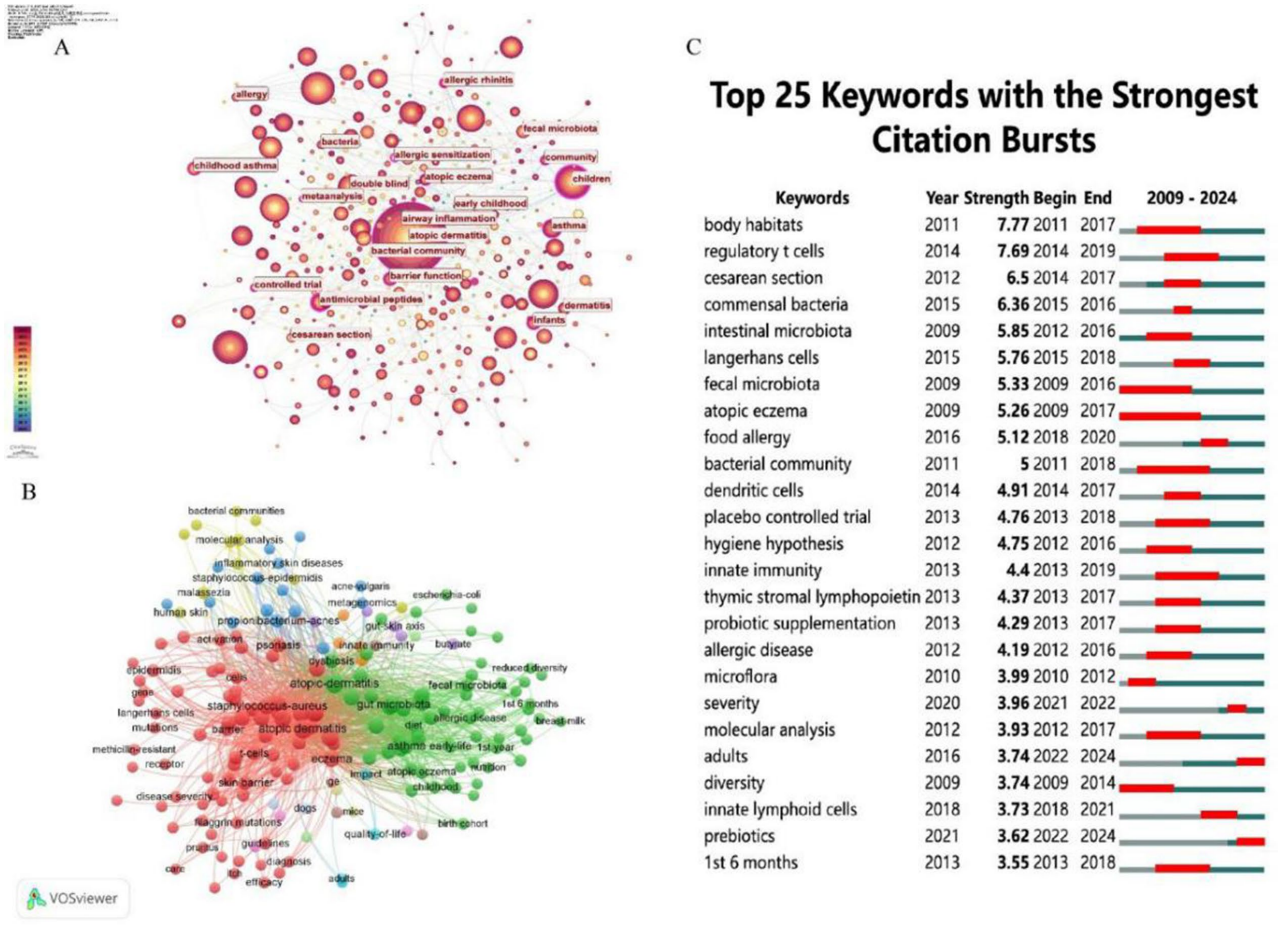
**(A)** The analysis of research fields and keywords concerning AD and microbiota. **(B)** Keyword co-occurrence network graph, node size reflects frequency of occurrence, color represents thematic clustering (e.g., red = skin microbiota, green = gut microbiota). **(C)** The top 25 keywords with the highest citation frequency burst.

The Table 6 lists the top 20 keywords by occurrence and their total link strength, indicating their frequency and connectivity in the literature. “Atopic dermatitis” (766 occurrences, 6,421 link strength) and “microbiome” (323 occurrences, 2,754 link strength) are the most frequent and highly connected, underscoring their centrality in the field. “Skin microbiome” (193 occurrences, 1,553 link strength) and “gut microbiota” (149 occurrences, 1,322 link strength) also rank high, highlighting the dual focus on skin and gut microbial ecosystems. The analysis of these figures (Figures 8, 9) and tables reveals that microbiome research, particularly in skin and gut contexts, has evolved significantly from 2009 to 2024. Early research (2009–2014) established the role of microbial diversity and composition in diseases like atopic dermatitis and eczema, driven by foundational studies on “microbiome” and “fecal microbiota.” The mid-period (2015–2019) saw a peak in citation bursts, focusing on microbial communities, immune responses, and clinical trials. In the most recent phase (2020–2024), research has shifted toward topics such as disease severity, adult populations, and dietary interventions like prebiotics—reflecting a more mature and diversified research landscape. The persistent centrality of “atopic dermatitis,” “gut microbiota,” and “*staphylococcus aureus*,” alongside emerging themes like the gut-skin axis and prebiotic therapies, underscores the field’s ongoing relevance and dynamic growth in understanding microbiota-host interactions and their therapeutic potential. Figure 9C lists keywords with the strongest citation bursts from 2009 to 2024, including the year of peak strength, start year, and end year of the burst. Furthermore, the highest burst strengths (e.g., 7.77 for “body habitats,” 7.69 for “regulatory T cells”) suggest these keywords had significant impact during their burst periods, driving research attention.

**Table 6:** Top 20 keywords with the highest occurrence times and their total link strength.

Rank	Keyword	Occurrences	Total link strength
1	Atopic dermatitis (atopic-dermatitis)	776	6,421
2	Microbiome	323	2,754
3	Skin microbiome	193	1,553
4	Children	177	1,577
5	Eczema	169	1,496
6	Gut microbiota	149	1,322
7	Diversity	138	1,130
8	*Staphylococcus-aureus*	138	1,172
9	Skin	137	1,120
10	Disease	129	1,088
11	Asthma	113	1,063
12	Double-blind	109	988
13	Probiotics	106	1,021
14	Inflammation	105	960
15	Intestinal microbiota	101	965
16	Microbiota	99	969
17	Colonization	97	797
18	Food allergy	83	827
19	Expression	80	644
20	*Staphylococcus aureus*	77	684

### Analysis of hotspots and frontiers

**Figure 10:**
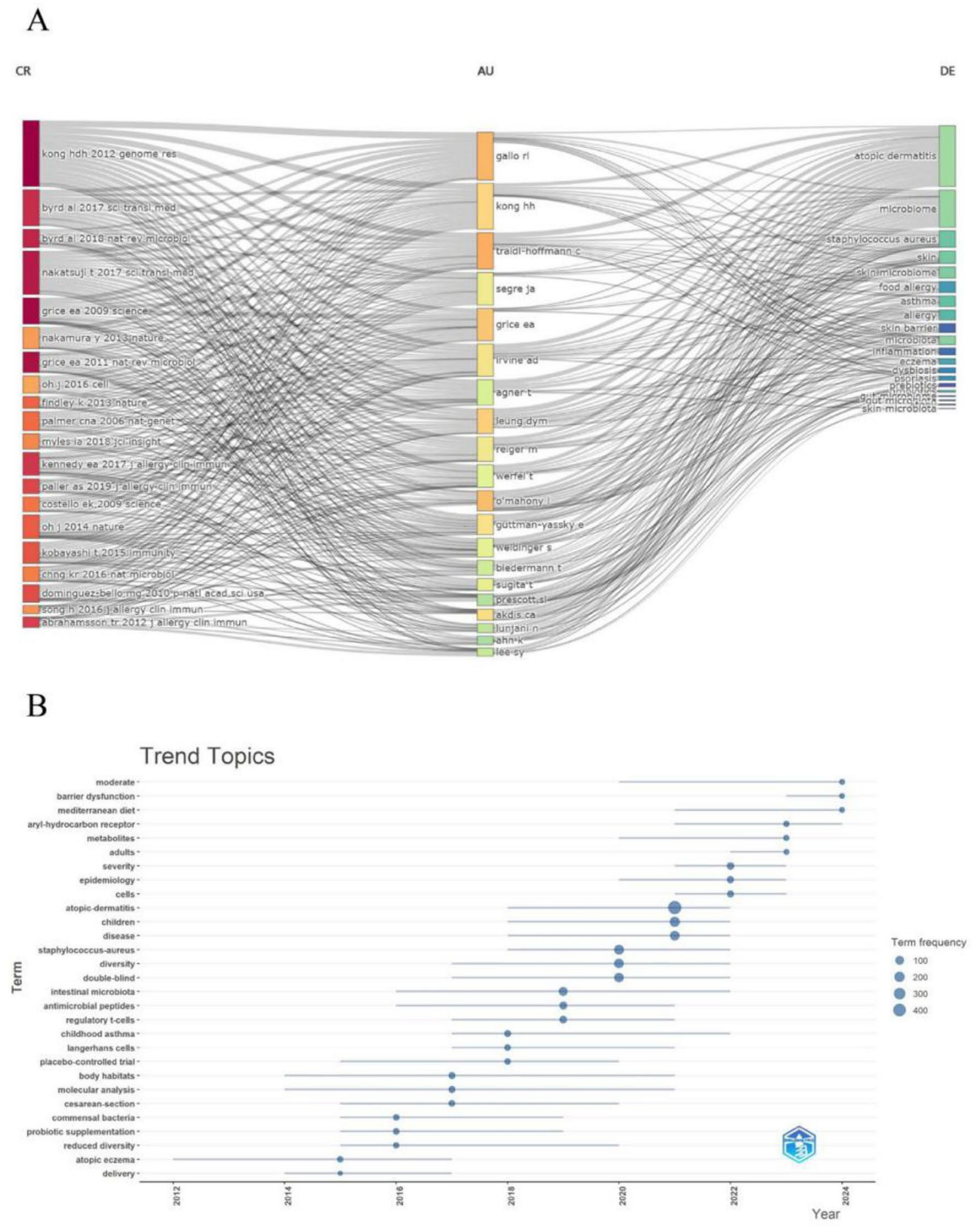
**(A)** This figure displays dynamic associations between authors (AU), references (CR), and keywords (DE), with color coding possibly indicating temporal or thematic evolution. The flow lines (streamlines) represent the strength and changes in connections between keywords, authors, and references. **(B)** This figure shows keyword trends from 2012 to 2024, with the x-axis representing years, the y-axis listing keywords, and dot size and color indicating keyword frequency (100–400 occurrences).

Overall, the frequency of keywords such as “adults,” “severity,” and “prebiotics” increased from 2021 to 2024, aligning with citation bursts (e.g., “adults” from 2022 to 2024, “prebiotics” from 2022 to 2024), which suggests a research shift toward disease outcomes, adult populations, and novel interventions. Meanwhile, keywords like “atopic dermatitis” and “*staphylococcus aureus*” have maintained consistently high frequencies, underscoring their enduring significance, while the rising prominence of “prebiotics” and “adults” highlights emerging trends in the field. The most significant research hotspot centers on the skin and gut microbiomes. All data and figures consistently show that the skin microbiome (e.g., “atopic dermatitis,” “eczema,” “*staphylococcus aureus*,” “skin barrier”) and the gut microbiome (e.g., “gut microbiota,” “fecal microbiota,” “diet”) are core areas of focus. This is further supported by the dense flow lines in Figure 10A and the high-frequency keywords in Figure 10B (e.g., “atopic dermatitis” with 400 occurrences, “microbiome” with 300 occurrences). Research intensity on these themes peaked between 2009 and 2019, with a notable surge in 2015, as evidenced by citation bursts and keyword frequencies. In the field of inflammatory and allergic diseases, conditions such as atopic dermatitis, eczema, asthma, and food allergy remain central, closely associated with “*staphylococcus aureus*” and “skin barrier.” Research on immunity and interventions, reflected in keywords like “innate immunity,” “regulatory T cells,” “probiotics,” and “prebiotics,” reveals a growing emphasis on microbial regulation of the immune system and its potential therapeutic applications. According to Figure 10B, “probiotics” (linked to probiotic supplementation) exhibited high frequencies from 2012 to 2018, while “prebiotics” gained prominence from 2021 to 2024, illustrating the evolving landscape of intervention research. These emerging and persistent themes underscore the importance of further exploring microbiota-based approaches in the treatment of atopic dermatitis and related conditions.

## Discussion

### General information

Based on the provided bibliometric data we made, our study identifies “atopic dermatitis” as the central theme of the research field, with key focus areas including the “skin microbiome,” “children,” “eczema,” and “gut microbiota.” The USA dominates in research output (360 publications) and impact (24,655 citations), establishing itself as a global leader, while the University of Copenhagen emerges as the most representative institution, contributing significantly to the field. The Journal of Allergy and Clinical Immunology, a top-tier journal, published 48 high-impact articles, amassing 5,561 citations. Among authors, Richard L. Gallo leads with 17 publications and 2,526 citations, whereas Heidi H. Kong’s 2012 genomic study (385 citations) stands out as one of the most influential references in the field. These findings underscore the rapid development and expanding scope of this research area over the past decade. They highlight the critical role of the microbiome–AD interplay and reveal a globally collaborative research network, with the United States at its core.

### Research trends and emerging topics

The dynamic evolution of research on AD and microbiota, as revealed through this bibliometric analysis, underscores the importance of tracking key terms and prominent themes to capture the latest advancements and anticipate future directions in this rapidly expanding field. By systematically examining publication outputs, citation patterns, and keyword trajectories from 2009 to 2024, we have identified critical research hotspots that reflect both foundational knowledge and emerging frontiers. This approach not only highlights quantitative trends—such as the post-2016 surge in publications and the dominance of skin and gut microbiome studies—but also provides a valuable lens through which to explore the cutting-edge developments that are shaping the future of AD research. Although research interest in adult-onset AD is increasing, there is still a lack of microbiome research in both adult onset and pediatric persistent a’d. Specifically, there is an urgent need for further exploration in areas such as the systematic comparison of microbial composition and function between the two, differences in immune system regulation by microbial metabolites, response characteristics of adult patients to microbial intervention, interaction patterns between skin microbiota and epidermal lipids, as well as microbial biomarkers and precision treatment strategies for adult AD. Future research should prioritize filling these gaps to promote the development of individualized intervention programs for adult AD.

In this context, we explore three pivotal areas: the influence of gut microbiota on AD pathogenesis via the gut–skin axis; the therapeutic potential of probiotics, prebiotics, and postbiotics in modulating AD symptoms; and the emergence of personalized treatment strategies informed by multi-omics technologies and individual microbiota profiles. These topics, grounded in the data-driven insights from our analysis, underscore the field’s progression toward a deeper mechanistic understanding and clinical innovation. They also illustrate how broad research trends are now converging into specific, actionable directions, bridging basic science with translational applications.

### The impact of gut microbiota on AD

In recent years, a growing body of research has highlighted the influence of gut microbiota on atopic dermatitis (AD) through the gut–skin axis, which involves complex mechanisms such as immune regulation, skin barrier function, and microbial metabolites (Ni et al., 2022; Zhang X.-E et al., 2024). The gut–skin axis is a bidirectional communication network linking intestinal microbiota with skin health and is now recognized as a crucial factor in the pathogenesis of AD (Martínez et al., 2021). Dysbiosis of the gut microbiota is believed to contribute to both the onset and progression of AD through multiple pathways (Song et al., 2016). One key pathway is immune system modulation (Chun et al., 2021). The gut microbiota helps regulate the host immune system, partly through the production of short-chain fatty acids (SCFAs) like butyric acid, propionic acid, and acetic acid, which are significant microbial metabolites (Trompette et al., 2014). For instance, a study by Wrześniewska et al. found that SCFAs can reduce skin inflammation and enhance skin barrier function by promoting regulatory T cell (Treg) activity and suppressing pro-inflammatory cytokines such as IL-6 and TNF-*α*. This mechanism is particularly important in pediatric AD, highlighting the role of gut microbiota in maintaining immune homeostasis (Wrześniewska et al., 2024). In addition to immune modulation, gut microbiota also influences AD by affecting the integrity of the skin barrier (Aguwa et al., 2023). Research suggests that microbial imbalance may reduce the expression of key epidermal barrier proteins such as filaggrin, thereby increasing skin permeability and triggering inflammation (Wang L. et al., 2022; Aguwa et al., 2023). Zhang et al., in their study of Chinese children with AD, found that gut microbiota alterations may disrupt skin barrier homeostasis through the gut–skin axis, although the precise mechanisms—such as cytokine signaling or T cell dynamics—require further clarification (Zhang X. et al., 2024). Currently, changes in microbial community composition are recognized as a central focus in AD research (Ganju et al., 2016; Díez-Madueño et al., 2025). Liu et al. conducted 16S rRNA gene sequencing to compare gut microbiota between AD patients and healthy controls in the Chinese population, identifying *Bacteroidaceae* and *Porphyromonadaceae* as potential microbial biomarkers for AD diagnosis (Ye et al., 2021). International studies support these findings, reporting that shifts in the relative abundance of bacteria such as *Escherichia coli* and *Clostridium* may correlate with AD severity (Hu et al., 2021; Kim J. et al., 2021). However, the exact causal relationships remain to be definitively established.

**Figure 11:**
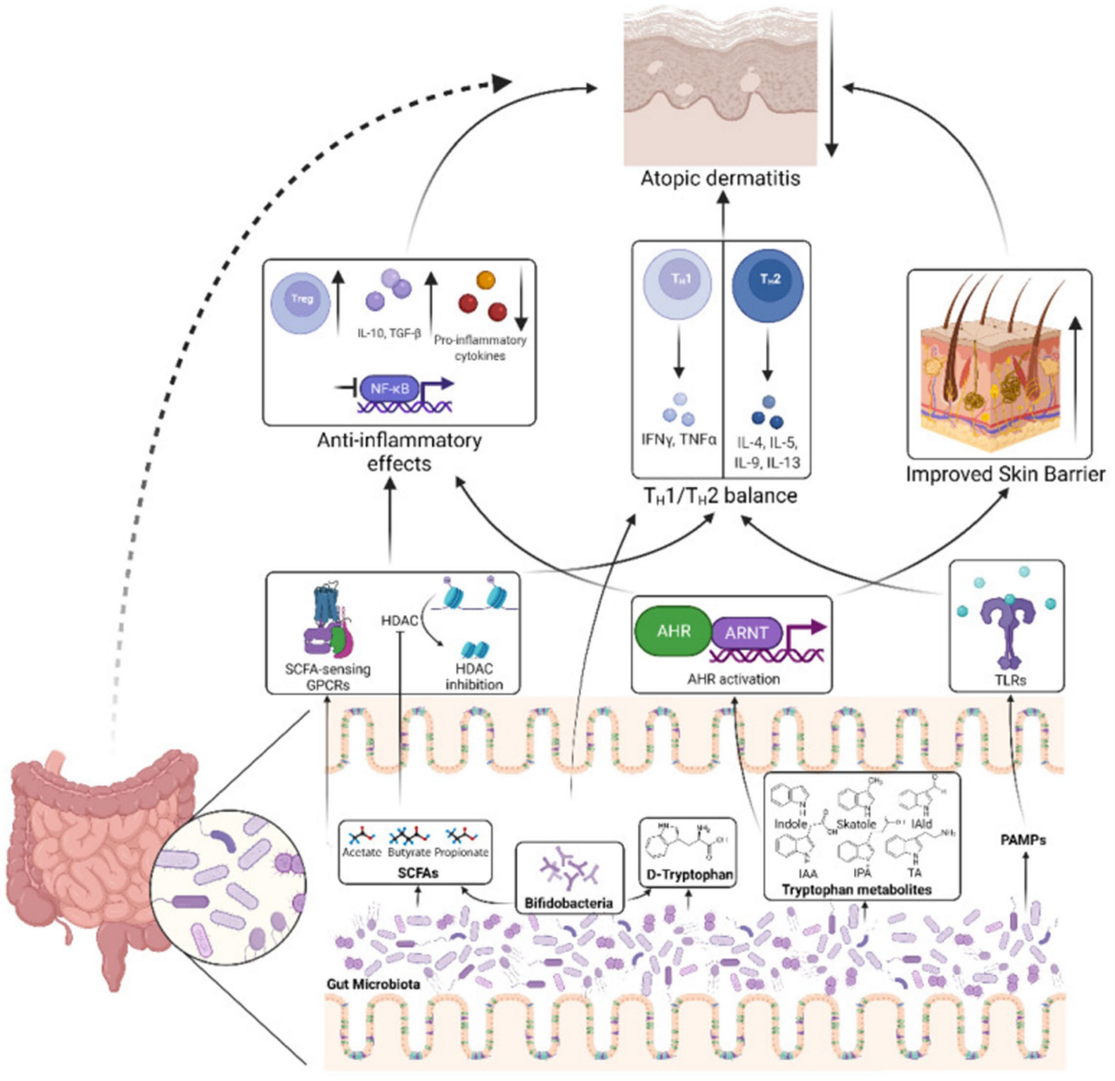
Mechanisms by which the gut microbiota influence AD pathogenesis. The gut microbiota produces SCFAs that can stimulate SCFA-sensing G-protein coupled receptors (GPCRs) or suppress histone deacetylases (HDACs), triggering downstream signaling pathways that reduce inflammation and help rebalance the TH1/TH2 immune response. Another microbial metabolite, D-tryptophan, also contributes to restoring the TH1/TH2 equilibrium. Bifidobacteria, a bacterial genus commonly found in probiotics, serve as a key producer of these beneficial metabolites. Additionally, tryptophan-derived microbial metabolites, such as indole-3-aldehyde (IAld), indole-3-acetic acid (IAA), indole-3-propionic acid (IPA), and tryptamine (TA), can activate the aryl hydrocarbon receptor (AHR) and its nuclear translocator (ARNT), leading to reduced inflammation and enhanced epidermal barrier function in the skin. Furthermore, pathogen-associated molecular patterns from the gut microbiota can engage toll-like receptors (TLRs), promoting the restoration of TH1/TH2 balance. Together, these processes contribute to alleviating AD. IAld, indole-3-aldehyde; IAA, indole-3-acetic acid; IPA, indole-3-propionic acid; TA, tryptamine; ARNT, aryl hydrocarbon receptor nuclear translocator. Reproduced from Alam, Md Jahangir et al. Manipulating Microbiota to Treat Atopic Dermatitis: Functions and Therapies. Pathogens (Basel, Switzerland). Copyright © 2022 by Alam et al. (2022).

The gut microbiota is involved in the occurrence and development of AD through various pathways, such as regulating the immune system, producing metabolites (such as SCFAs), and interacting with the skin microbiota (Ito and Amagai, 2022; Zhang X.-E et al., 2024). However, it is worth noting that there is some inconsistency in the research results regarding the immunomodulatory effects of SCFAs (Sasaki et al., 2024). This difference may be related to various methodological factors such as sample type (such as feces, serum), collection time, analytical methods (such as GC–MS, LC–MS), pretreatment steps, internal standard selection, statistical methods, etc. (Chalova et al., 2023; Xiao et al., 2023). In addition, differences in dietary habits, disease status, age, and other factors of the research subjects can also lead to fluctuations in SCFA levels. Therefore, future research should standardize sample collection, preprocessing, instrument parameters, internal standard use, and data processing to improve comparability between studies and reproducibility of results (Trompette et al., 2022). In addition, future research needs to further elucidate the specific mechanism of action between gut microbiota and AD, optimize intervention measures, and provide scientific basis for precise prevention and treatment of AD (Figure 11).

### The use of probiotics, prebiotics, and prebiotics on AD

With the deepening of research on gut microbiota, intervention strategies targeting microbial modulation—such as probiotics, prebiotics, and synbiotics—are gaining increasing attention for their potential in the treatment of AD (Wang Y. et al., 2022; Lee et al., 2024). Probiotics refer to live probiotic microorganisms, such as *Lactobacillus* and *Bifidobacterium*, that function by regulating the gut skin axis (Mahmud et al., 2022). Numerous studies have demonstrated that probiotics can alleviate skin inflammation and enhance skin barrier function in AD patients by promoting the activity of regulatory T cells (Tregs) and suppressing the production of pro-inflammatory cytokines, including IL-6 and TNF-*α* (Mahmud et al., 2022; Emokpae et al., 2024). Meanwhile, much high-quality clinical evidence-based medical evidence have also confirmed this (Wu et al., 2017; Carucci et al., 2022). A recent meta-analysis revealed that probiotic supplementation significantly reduced the incidence of AD in children, with a relative risk (RR) of 0.75 (95% CI: 0.62–0.91), and also led to a reduction in disease severity as measured by the SCORAD index, with a weighted mean difference (WMD) of −5.2 (95% CI: −7.1 to −3.3) (Wang and Xu, 2025). However, the efficacy of specific probiotic strains varies across studies. For example, a 2023 meta-analysis found that *Lactobacillus salivarius* showed the most pronounced effect (RR: -9.79, 95% CI: −13.04 to −6.54), particularly in adults with severe AD. *Lactobacillus acidophilus* also demonstrated measurable benefits (RR: -5.77, 95% CI: −10.82 to −0.72) (Husein-ElAhmed and Steinhoff, 2023). Moreover, the research results are controversial, such as the limited effectiveness of probiotics in infants under 6 months old, which may be related to the immature gut microbiota (Vael and Desager, 2009). In addition, insufficient differentiation between AD and other allergic diseases (such as asthma) in different studies affects the specificity of conclusions.

Postbiotics are microbial metabolites—such as short-chain fatty acids (SCFAs), including butyric acid, propionic acid, and acetic acid—that function by modulating host immune responses and enhancing skin barrier integrity (Kreouzi et al., 2025). A study by Husein Husein El Ahmed et al. demonstrated that supplementation with endogenous metabolites significantly reduced the severity of AD, with a weighted mean difference (WMD) of −4.8 (95% CI: −6.5 to −3.1), showing particular effectiveness in patients with gut microbiota dysbiosis (Husein-ElAhmed and Steinhoff, 2023). In addition, preliminary clinical trials have shown that oral administration of epigenetic preparations can improve skin barrier function and reduce itching (Li and Yosipovitch, 2020). In addition, the local application of epigenetic cream has been shown to reduce skin inflammation and bacterial colonization (Chen et al., 2023; Isidro-Hernández et al., 2023). The underlying mechanism may involve SCFAs interacting with G protein-coupled receptors—such as GPR41 and GPR43—to regulate immune responses, enhance regulatory T cell (Treg) activity, and suppress the release of pro-inflammatory cytokines.

**Figure 12:**
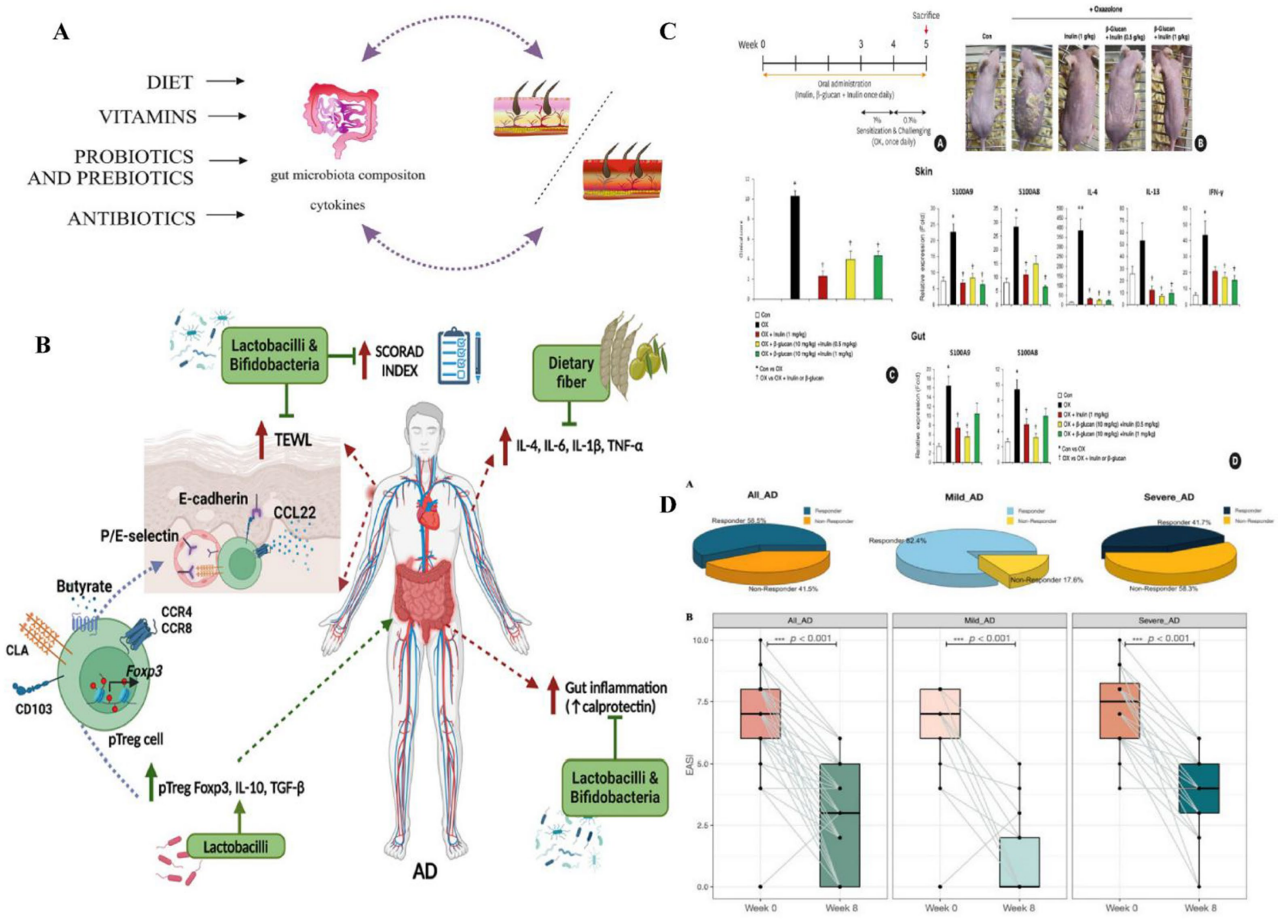
**(A)** The effects of diet, vitamins, probiotics, prebiotics, and antibiotics on the regulation of contact hypersensitivity by modulating the gut microbiota. Diet, vitamins, probiotics, prebiotics, and antibiotics can promote or inhibit skin diseases, including AD. Reproduced from Kiecka, Aneta et al. Modulation of allergic contact dermatitis via gut microbiota modified by diet, vitamins, probiotics, prebiotics, and antibiotics. *Pharmacological reports: PR* vol. 75, 2 (2023): 236–248. Copyright © 2020 by the authors Kiecka et al. (2023). **(B)** Role of prebiotics and probiotics in AD through Treg cells and cytokines. Prebiotics and probiotics lower gut and systemic inflammation, boost anti-inflammatory cytokines, and enhance Treg cell differentiation. Peripheral Treg (pTreg) cells with Foxp3 express skin-homing markers (CLA, CCR4/8) and retention marker CD103, migrating to the dermis to reduce AD inflammation. This improves the skin barrier and lowers the SCORAD index. TNF, tumor necrosis factor; TGF, transforming growth factor; CLA, cutaneous lymphocyte-associated antigen; TEWL, transepidermal water loss. Reproduced from Rios-Carlos, Marcela et al. Unraveling the gut-skin axis in atopic dermatitis: exploiting insights for therapeutic strategies. *Gut microbes.* Copyright © 2024 by Rios-Carlos et al. (2024). **(C)** The preventive effect of prebiotics on OX induced AD mice: (A) Experimental design schematic diagram showing the timeline of oral administration of prebiotics and OX sensitization; (B) Images of skin lesions on the back of mice were compared between the control group (Con), OX group, and prebiotic pretreatment group (OX + inulin, OX+*β* – glucan + inulin); (C) Clinical score (degree of skin lesions); (D) The mRNA levels of S100A8, S100A9, and inflammatory cytokines in the skin and intestine indicate that prebiotics reduce the expression of inflammatory markers. Probiotics (such as inulin and *β*- glucan) alleviate OX induced AD symptoms by reducing skin lesions in AD mice, lowering the expression of inflammatory cytokines (IL-4, IL-13, IFN -*γ*) and calprotectin (S100A8, S100A9), inhibiting epidermal thickening and immune cell infiltration, and protecting skin barrier proteins (such as loricin and filaggrin). Reproduced from Kang, Minje et al. Therapeutic and Preventive Effect of Orally Administered Prebiotics on Atopic Dermatitis in a Mouse Model. *Allergy, asthma & immunology research* vol. Copyright © 2023 by Kang et al. (2023). **(D)** Probiotic mixture significantly ameliorates AD severity. (A) Distribution of responders and non-responders among All-AD, mild AD, and severe AD patients. (B) Alteration of the AD severity score before and after novel E3 probiotics mixture administration among all AD, mild AD, and severe AD patients. Denoted *p* < 0.001. Reproduced from Wang, Yiwei et al. “Effect of a Novel E3 Probiotics Formula on the Gut Microbiome in Atopic Dermatitis Patients: A Pilot Study.” *Biomedicines.* Copyright © 2022 by Wang Y. et al. (2022).

To date, research on epigenetic factors in the context of AD remains limited, and literature published up to 2025 has yet to provide sufficient clinical trial data (Zhao et al., 2020; El-Salhy et al., 2022). Further studies are needed to validate the long-term safety and efficacy of epigenetic interventions. Additionally, Liu et al. suggested that certain epigenetic elements may be associated with specific bacterial taxa, such as *Bacteroidetes*; however, the causal relationship between them remains unclear (Ye et al., 2021). Although these intervention strategies show considerable potential, several challenges persist. First, therapeutic efficacy can vary depending on factors such as age group, selection of strains or ingredients, and individual microbiota composition. For instance, probiotics may demonstrate greater effectiveness in adults than in children, although current research findings are inconsistent. Second, there is a lack of comprehensive research on optimal dosage, treatment duration, and the appropriate timing for intervention. As a result, the development of personalized treatment strategies is urgently needed. Finally, as epigenetics is still an emerging area within AD research, clinical evidence remains scarce. More high-quality, randomized controlled trials are necessary to support its clinical application (Figure 12).

### The intervention of microbial transplantation

**Figure 13:**
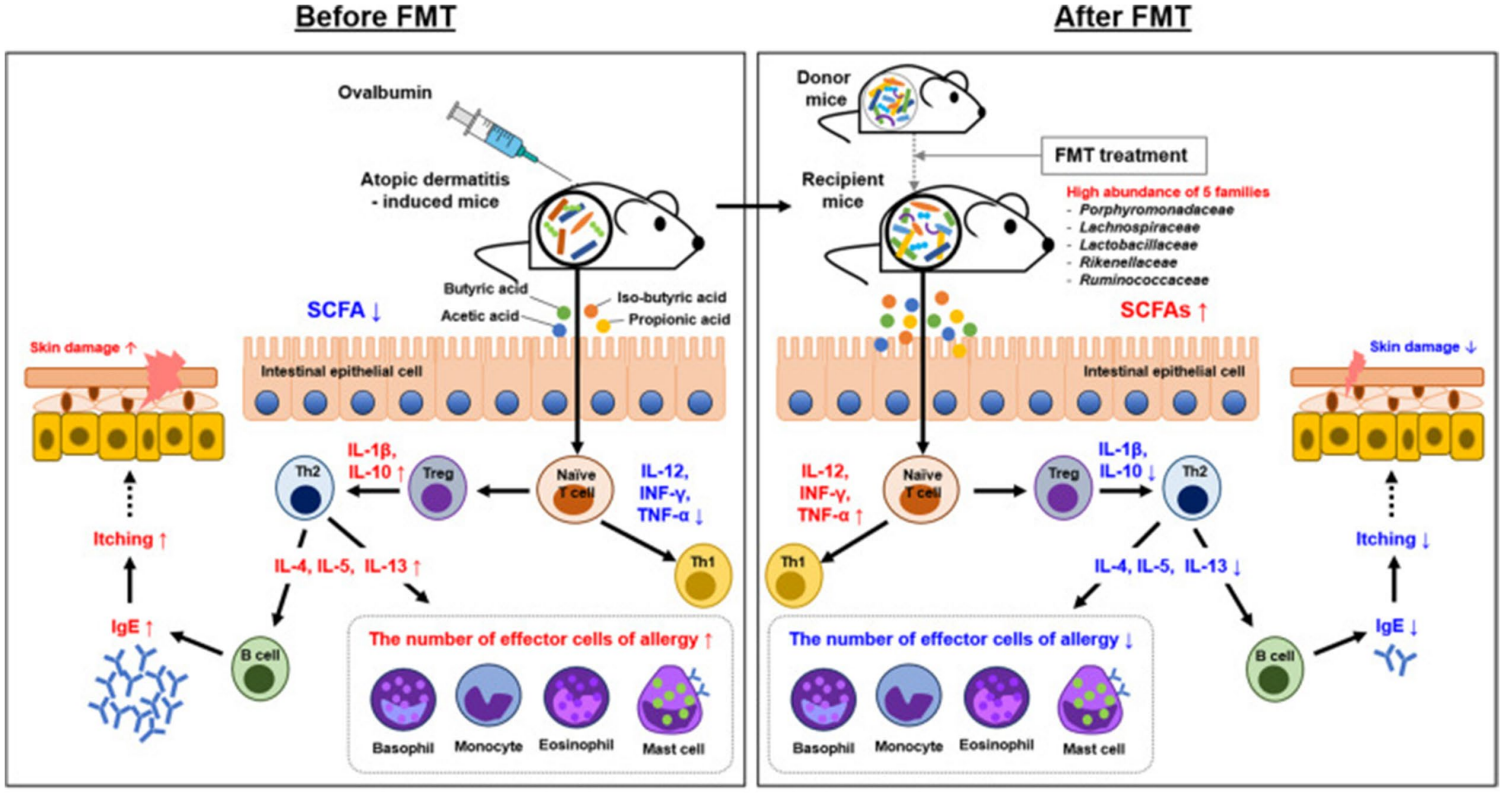
Overview of the mechanism underlying the effect of FMT in AD. The mechanism involved in AD control was proposed by FMT and gut microbiota. Mechanistic links are indicated by solid black lines (—, direct links) and dotted black line (—, speculative link). Reproduced from Kim, Jong-Hwa et al. Gut microbiota restoration through fecal microbiota transplantation: a new atopic dermatitis therapy. Experimental & molecular medicine. Copyright © 2021 by Kim J.-H et al. (2021).

Fecal microbiota transplantation (FMT) typically involves transplanting the fecal microbiota of a healthy donor into the patient’s gut (Liu et al., 2024), with the aim of restoring the diversity and function of the gut microbiota, thereby affecting AD through the gut dermal axis (Mashiah et al., 2022). A randomized, double-blind, controlled trial conducted in 2024 evaluated the efficacy and safety of FMT in adults with moderate to severe AD. The results showed that patients in the FMT group exhibited significant improvements in Eczema Area and Severity Index (EASI) scores, with a higher proportion achieving EASI-50 (a 50% reduction in EASI score) compared to the placebo group. Importantly, no serious adverse events were reported (Liu et al., 2024). The study also found that FMT altered the composition of the gut microbiota, increased the abundance of *Megamonas fusiformes*, and regulated immune responses by reducing the proportion of Th2 and Th17 cells, decreasing serum TNF-*α* and total IgE levels (Liu et al., 2024). In addition, FMT may enhance the production of short-chain fatty acids—such as butyric acid—thereby improving skin barrier function and reducing inflammatory responses, a mechanism supported by mouse model findings in the study by Jiang et al. (2023). Interestingly, Liu et al. and other randomized controlled trials have found that FMT also influences microbial functional pathways, such as changes in the biosynthesis of 1,4-dihydroxy-6-naphthoate II, suggesting that metabolic regulation may also be involved, although the precise mechanisms require further elucidation (Liu et al., 2024). At present, our exploration of microbiota transplantation has also shown potential, especially wash microbiota transplantation (WMT), an improved FMT method that reduces the risk of infection by purifying donor feces (Davidson et al., 2019). A recent case report evaluated the efficacy of WMT in severe AD patients in adolescents, and the results showed a significant decrease in SCORAD scores, improvement in gut and skin microbiota, and no significant safety issues (Deng et al., 2023). Although the convenience and safety of WMT have received attention in research, the existing study sample size is only individual cases and lacks large-scale clinical data, which limits its promotion (Chen et al., 2022; Wu et al., 2023). We look forward to large-scale clinical studies in the future to explore the application scope of WMT. In order to optimize the effectiveness of FMT in AD, strict donor screening criteria need to be established in future research, including: selecting healthy donors with high diversity of gut microbiota and abundant abundance of *Bacteroides*, *Bifidobacterium*, *Fecal bacteria*, etc. (Yang et al., 2023). Exclude donors carrying potential pathogenic bacteria and resistance genes (Yockey et al., 2022); Individualized donor matching is performed based on the gut microbiota and metabolic characteristics of AD patients, such as SCFAs levels and Clostridium abundance (Liang et al., 2024); Select donors with immune regulatory ability (rich in *Lactobacillus* and other microbial communities) and stable intestinal metabolic characteristics (Wu et al., 2023). Through this strategy, FMT is expected to achieve longer lasting efficacy and reduce the risk of recurrence (Yang et al., 2023; Liang et al., 2024). In addition, it is suggested that future research should focus on matching donor screening criteria with AD patient subtypes to improve the accuracy and sustainable efficacy of FMT intervention (Figure 13).

Although both FMT and WMT demonstrate therapeutic potential, their efficacy varies depending on individual microbiota composition and disease severity. For instance, a clinical trial conducted in 2025 reported limited persistence of immune regulation in some patients during follow-up, indicating a potential risk of disease recurrence (Kenney et al., 2025). An international study involving adult AD patients showed an average SCORAD score reduction of 59.2%; however, while 77% of patients achieved a 50% improvement, only 44% reached a 75% improvement, underscoring the variability in response (Mashiah et al., 2022). Recent researches emphasize the potential of WMT in adolescents, but there is controversy due to the small sample size and difficulty in promoting it (Bourrain et al., 2013; Deng et al., 2023). The current existing research mainly focuses on adult patients, and the efficacy of FMT in children with AD is still unclear, requiring more clinical trials (Zou et al., 2022). Optimizing transplantation plans, such as screening criteria for donor microbiota characteristics (such as abundance of *Bacteroidetes* and *Clostridium* in healthy adults), is a future research focus (Wang X.-Z et al., 2024). In addition, the long-term efficacy and personalized treatment strategies of FMT need further exploration (Gulati et al., 2023). The research limitations include small sample size, lack of multi center experimental data, and insufficient depth of mechanism research (Zhao et al., 2023; Liu et al., 2024). In the future, it is necessary to combine metagenomic shotgun sequencing and other technologies to deeply analyze the functional changes of microbial communities.

### Precision medicine: personalized treatment on AD patient

With the rapid development of precision medicine and microbiology, personalized therapy has become an emerging strategy that aims to improve treatment effectiveness and reduce side effects by designing intervention plans based on individualized microbiota characteristics of patients (Kamal et al., 2023). At present, the latest research, combined with multi omics analysis and clinical translation, provides important evidence for microbiota intervention based on individual characteristics, especially in the phenotype recognition and treatment strategy optimization of AD (Gates et al., 2022; Meng et al., 2024). This section will explore in detail the scientific foundations, clinical translational potential, and future directions of personalized therapy in the context of microbiota-based treatments.

**Figure 14:**
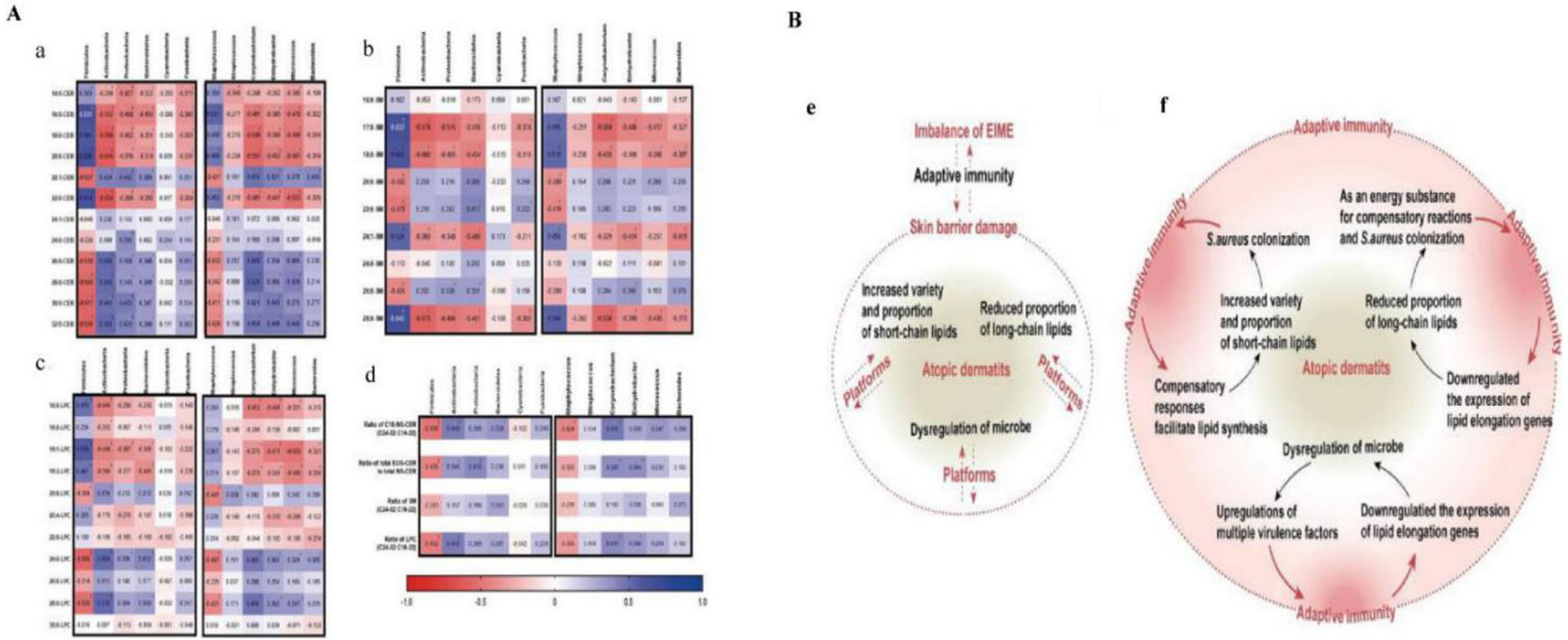
**(A)** A heat map depicts the relationships between the skin microbiome, categorized at the phylum and genus levels, and various lipid components, such as the proportions of NS ceramides (a) (ceramides containing C18 sphingosine and nonhydroxy fatty acids), sphingomyelins (b), lysophosphatidylcholine (c), and the ratios of long-chain to short-chain fatty acids (d). This visualization is based on skin samples from individuals with atopic dermatitis and healthy controls, with Spearman correlation coefficients used to measure the strength of these associations. **(B)** The epidermal lipid-microbiome loop and its pivotal role in immunity in AD. (e) Disruptions in the positive feedback cycle between epidermal lipids and the skin microbiome significantly contribute to the dysregulation of the epidermal immune microenvironment (EIME) in AD. These disturbances manifest as a weakened lamellar membrane, heightened susceptibility to skin pathogens, and shifts in the functionality of both innate and adaptive immune systems. (f) In AD, the interaction between the epidermal lipid-microbiome loop and immune responses is profound. Within the EIME, two immune-mediated strategies enhance the skin barrier: one involves supplementation through epidermal lipids, and the other entails sophisticated regulation of the microbial community. These actions activate adaptive immunity, which subsequently perpetuates the irregular lipid composition, promotes the proliferation of skin pathogens, and intensifies the inflammatory response characteristic of AD. Reproduced from Wu, Junchao et al. The epidermal lipid-microbiome loop and immunity: Important players in atopic dermatitis. Journal of advanced research. Copyright © 2025 by Wu et al. (2025).

The application of multi-omics technologies enables the identification of distinct phenotypes, microbiota profiles, and host metabolic traits in atopic dermatitis (AD), providing a foundation for personalized therapeutic strategies. For example, Lee et al. employed a multi-omics approach—including microbiota analysis, metabolomics, and intestinal epithelial transcriptomics—to study 2,247 children from the COCOA (Cohort for Childhood Origin of Asthma and Allergic Diseases) birth cohort. They identified five distinct AD trajectories: never/rare, early transient, mid transient, late-onset, and early persistent. Notably, the early persistent phenotype was associated with reduced abundance of *Ruminococcus gnavus*, lower acetic acid levels, involvement of ACSS2 and Janus kinase–STAT signaling pathways, and systemic Th2 inflammation. In contrast, the late-onset phenotype was linked to IL-17 activity and skin barrier dysfunction. These findings highlight the potential of individual microbiota and metabolic profiles to serve as biomarkers for precision medicine and personalized intervention strategies (Lee et al., 2025). Wu et al. further revealed the role of the positive feedback loop between epidermal lipids and microbiota in AD, proposing that lipid composition abnormalities and microbiota dysbiosis drive disease through immune regulation. The skin of AD lesions shows a decrease in CER EOS and long-chain fatty acids, an increase in short chain fatty acids and CERs, accompanied by opportunistic pathogen colonization (such as *Staphylococcus aureus*) (Wu et al., 2025). These changes can be improved by supplementing epidermal lipids or intervening with probiotics, emphasizing the possibility of personalization based on individual lipid microbiota characteristics (Figure 14A) (Wu et al., 2025). Fyhrquist et al. introduced the concept of AD endotypes, emphasizing the genetic and immunological heterogeneity among patients. They highlighted the strong association between a Th2-dominant phenotype and microbiota dysbiosis, suggesting that multi-omics tools—such as 16S rRNA sequencing—can be employed to identify personalized microbiota signatures. These insights may inform microbiota-targeted therapies, including FMT and epigenetic interventions such as short-chain fatty acid supplementation (Fyhrquist et al., 2025).

The key to personalized treatment lies in designing intervention strategies based on AD phenotype and microbiota composition (Kreouzi et al., 2025). For example, for early-stage persistent AD patients, priority can be given to supplementing acetic acid producing bacteria (such as *Ruminococcus gnavus*) or using probiotics that regulate Th2 inflammation (such as *Lactobacillus rhamnosus*) to restore gut microbiota balance (Kalashnikova et al., 2024; Dera et al., 2025). Wu et al.’s research supports interventions targeting patients with EIME imbalance through lipid supplementation (such as CER EOS) combined with probiotics, particularly effective in individuals with impaired skin barrier function (Wu et al., 2025). In addition, we found that the epidermal lipid microbiome loop and immunity play key roles in AD (Wu et al., 2025). Barrier-directed treatments, such as lipid replacement combined with complex microbial regulation, may influence adaptive immune responses and modulate inflammation in AD (Figure 14B). In line with this, a recent clinical trial on FMT demonstrated that donor selection based on recipients’ gut microbiota profiles significantly improved EASI scores, highlighting the promise of personalized FMT strategies (Kenney et al., 2025). Additionally, Tingting et al. evaluated the effects of combination therapy using topical antibiotics and corticosteroids versus corticosteroids alone, finding that treatment outcomes varied with individual skin microbiota profiles—indicating a need to further optimize personalized treatment plans (Tingting et al., 2025). A noteworthy finding is the association of early-persistent AD with intestinal acetic acid levels and *Ruminococcus gnavus* abundance, which challenges the traditional skin-focused treatment paradigm and underscores the importance of gut-targeted interventions (Barman et al., 2024). In clinical practice, this means that doctors may need to combine multiple omics data from the gut and skin to design more comprehensive personalized plans, such as using probiotics and lipid supplements simultaneously, especially in pediatric patients.

Although we have found significant progress in personalized differences and therapeutic effects of using microbiomes to treat AD, challenges include the lack of large-scale multicenter trials to validate the effectiveness of personalized regimens, as well as standardized methods for integrating genetic, microbiome, and clinical data. We believe that future research should focus on developing multi omics models based on artificial intelligence to predict individual responses to microbiota interventions and optimize the combination strategies of probiotics, prebiotics, and FMT to achieve maximum clinical translation. For example, Zhang et al. studied the role of pharyngeal microbiota in AD and found that differences in microbial composition may affect disease progression, suggesting that we can further explore the interaction between skin and gut microbiota (Zhang et al., 2025). Meanwhile, Liu K et al. discussed the interaction between skin environment and microbiota in immune related skin diseases, emphasizing the potential of microbiota modulation in AD, but pointing out the need for further research to deepen its therapeutic applications (Liu et al., 2025). The combination of clinical and basic research provides a solid foundation for the clinical translation of personalized treatment, but technical, cost, and standardization issues still need to be addressed. In the future, with the deepening of research and technological progress, personalized treatment is expected to become an important strategy for the management of AD, providing patients with more efficient and personalized treatment plans (Gatmaitan and Lee, 2023; Mesjasz et al., 2023). In addition, attention should be paid to the regulatory effect of exogenous lipids (such as moisturizers, skincare products, and ointments) on the lipid microbial cycle in the epidermis (Wu et al., 2025). Especially in the adolescent population, the symbiotic relationship between sebaceous gland development and skin microbiota is not yet fully mature (Yin et al., 2023). Exogenous lipids may affect the microbial community structure by altering sebum composition (such as reducing squalene, supplementing ceramides) or regulating sebum secretion (such as oil controlling skincare products), thereby interfering with the lipid microbial cycle and local immune balance (Ahlström et al., 2024; Schachner et al., 2024). Future research should systematically explore the regulatory effect of exogenous lipids on the AD microbiome in adolescents and different age groups to improve this model and guide personalized interventions.

### Limitations

This study, while comprehensive, encounters several limitations typical of bibliometric analyses and systematic reviews. In addition, this study only relied on WOSCC for literature analysis and did not cover literature in databases such as PubMed, Scopus, Embase, etc. This may result in omissions in clinical intervention studies, conference papers, and non-English literature, leading to biases in citation and trend analysis in specific fields such as probiotics and microbial transplantation. Future research will adopt a multi database strategy, combining quantitative and qualitative analysis to enhance the completeness and reliability of research results. Then, despite efforts to standardize data by consolidating keywords, author names, and institutional affiliations, inconsistencies in naming conventions (e.g., variations in spelling or institutional nomenclature) may have prevented full unification of identical entities, potentially affecting the accuracy of collaboration networks and citation metrics. Additionally, the bibliometric approach, while effective for mapping trends and hotspots, does not assess the quality or clinical impact of individual studies, which could limit insights into their practical applicability. Finally, the rapid evolution of AD and microbiota research means that some of the most recent developments, particularly those post-2024, may not be fully captured within the timeframe of this analysis (2009–2024), potentially underrepresenting emerging trends or cutting-edge interventions. In addition, the inconsistency of SCFA related research results is partly due to methodological differences, including differences in sample types, analytical techniques, preprocessing methods, and statistical analysis methods. To solve this problem, standardization of metabolomics technology, including sampling, detection, and data processing procedures, should be promoted in the future to improve the comparability and reliability of results. These limitations highlight the need for future studies to incorporate multi-database searches, broader linguistic inclusion, and qualitative assessments to enhance the robustness and scope of the findings.

## Conclusion

This study highlights the developmental trends and research hotspots in the field of atopic dermatitis (AD) and microbiota, offering valuable guidance for future scientific exploration and clinical practice. It is recommended that future studies include clinical trials investigating the efficacy of prebiotic interventions in adult AD patients, and consider the integration of such findings into public health strategies—for example, by promoting high-fiber diets as a preventive measure against AD. Microbiome research in AD holds tremendous potential, bridging fundamental biological mechanisms with practical clinical applications. Emerging focal areas include the pathogenic role of the skin microbiome—particularly *Staphylococcus aureus*; the influence of the gut–skin axis on systemic immune responses; and the impact of dietary interventions such as probiotics, prebiotics, and Mediterranean diets. These areas represent the cutting edge of current research and are already contributing to advances in precision medicine, personalized treatment approaches, and public health initiatives. Looking ahead, sustained multidisciplinary collaboration across microbiology, immunology, dermatology, and nutrition will be crucial for driving innovations in the prevention and management of AD. Such integrative efforts promise more effective therapeutic strategies and a better quality of life for patients.

## Data Availability

The original contributions presented in the study are included in the article/supplementary material, further inquiries can be directed to the corresponding authors.
